# Transcriptome-(phospho)proteome characterization of brain of a germline model of cytoplasmic-predominant Pten expression with autism-like phenotypes

**DOI:** 10.1038/s41525-021-00201-z

**Published:** 2021-06-02

**Authors:** Stetson Thacker, Charis Eng

**Affiliations:** 1 Genomic Medicine Institute, Lerner Research Institute, Cleveland Clinic, Cleveland, OH USA; 2 Department of Molecular Medicine, Cleveland Clinic Lerner College of Medicine, Case Western Reserve University, Cleveland, OH USA; 3 Center for Personalized Genetic Healthcare, Cleveland Clinic Community Care and Population Health, Cleveland, OH USA; 4 Department of Genetics and Genome Sciences, Case Western Reserve University School of Medicine, Cleveland, OH USA; 5 Germline High Risk Focus Group, CASE Comprehensive Cancer Center, Case Western Reserve University School of Medicine, Cleveland, OH USA

**Keywords:** Autism spectrum disorders, Proteomics

## Abstract

*PTEN* has a strong Mendelian association with autism spectrum disorder (ASD), representing a special case in autism’s complex genetic architecture. Animal modeling for constitutional *Pten* mutation creates an opportunity to study how disruption of Pten affects neurobiology and glean potential insight into ASD pathogenesis. Subsequently, we comprehensively characterized the neural (phospho)proteome of *Pten*^*m3m4/m3m4*^ mice, which exhibits cytoplasmic-predominant Pten expression, by applying mass spectrometry technology to their brains at two-weeks- (P14) and six-weeks-of-age (P40). The differentially expressed/phosphorylated proteins were subjected to gene enrichment, pathway, and network analyses to assess the affected biology. We identified numerous differentially expressed/phosphorylated proteins, finding greater dysregulation at P40 consistent with prior transcriptomic data. The affected pathways were largely related to PTEN function or neurological processes, while scant direct overlap was found across datasets. Network analysis pointed to ASD risk genes like Pten and Psd-95 as major regulatory hubs, suggesting they likely contribute to initiation or maintenance of cellular and perhaps organismal phenotypes related to ASD.

## Introduction

*PTEN* (Phosphatase and Tensin homolog on chromosome Ten, OMIM #601728), a well-studied, tumor suppressor gene, is known for its canonical role as the major antagonist of PI3K/AKT/mTOR signaling, a crucial growth and survival intracellular cascade, via the lipid modality of PTEN’s dual-specific phosphatase function^1–3^. Germline *PTEN* mutations are considered a rare cancer predisposition mechanism, while also representing one of the strongest Mendelian associations with autism spectrum disorder (ASD)^4,5^. Ostensibly, individuals with germline *PTEN* mutations present with a diverse array of clinical phenotypes, ranging from high risk of benign and malignant neoplasia to ASD^6^.

While there have been several rigorous efforts to explore the transcriptome of ASD^7–15^, there has been limited exploration of the proteome of ASD^16–19^. Little has been done to understand the proteome, let alone characterize the functional effectors of the phosphoproteome in syndromic ASD contexts, such as those arising from Mendelian gene variation, here, germline *PTEN* mutation. Thus, we believe that animal modeling for *PTEN* mutation-associated ASD followed by the deployment of proteomic/phosphoproteomic technologies could represent an excellent approach for disentangling the complexity associated with autism pathophysiology.

For insight into potential disease mechanisms of PTEN-ASD (i.e., persons with ASD carrying germline *PTEN* mutations), we generated the *Pten*^*m3m4*^ mouse model, which exhibits predominantly cytoplasmic Pten localization^20–22^. The *Pten*^*m3m4*^ mouse presents with clear neurological phenotypes; homozygous mutants have severe macrocephaly from increased brain mass, subsequent to neuronal hypertrophy, astrogliosis, microgliosis, and white matter overabundance^22–24^. Furthermore, we observed deficits in neuronal differentiation in neural stem cells (NSCs) derived from *Pten*^*m3m4*^ mice^25^. Despite these various pathologies, *Pten*^*m3m4/m3m4*^ mice have fairly normal learning and memory phenotypes. However, *Pten*^*m3m4/m3m4*^ mice display an aberrant increase in sociability among males, and both sexes have impaired motor coordination^22^. Most importantly, the neural transcriptome of these mice shares a significant portion of differentially expressed genes with the neural transcriptomes of individuals with idiopathic ASD, namely decreased expression of genes involved in synaptic functions and increased expression of genes related to neuroinflammation^26^. Moreover, there is a shift toward increased intron retention in the brains of these mice and dysregulation in microexon splicing^27^, a potential shared hallmark of autism pathophysiology^28^. Intriguingly, there is growing evidence suggesting mutations in *PTEN* that decrease nuclear localization (e.g., the M3M4 mutation) associate strongly with autism-like phenotypes^29^. Thus, the *Pten*^*m3m4*^ mouse is likely a useful model for exploring the neurobiology that is affected by germline mutations in *PTEN*.

Given the prior findings from the *Pten*^*m3m4*^ mouse and the lack of neural (phospho)proteomic data available to interrogate *PTEN* pathophysiology in the context of ASD, we performed proteomic, phospho-serine/threonine (pS/pT) proteomic, and phospho-tyrosine (pY) proteomic surveys of two-week-old (P14) hemibrains and six-week-old (P40) cortices of *Pten*^*m3m4/m3m4*^ mice compared to littermate wildtype samples–an experimental design shared by our prior work on the neural transcriptome^26^. We hypothesized that disruption of Pten, which has poorly characterized but likely promiscuous protein phosphatase function, would likely perturb the phosphoproteome in ways that contribute to pathophysiology that disrupts synaptic functions and promotes stress or neuroinflammatory processes. Furthermore, we anticipated disruption of the proteome given the previously documented changes in the transcriptome and anticipated relationships between the differential expression in the transcriptome and proteome. Ultimately, our transcriptome-proteome-phosphoproteome exploration of the molecular complexity of the *Pten*^*m3m4*^ brain has illuminated the variation within and between different –omic landscapes, identifying important candidate regulators and effectors of *PTEN* dysfunction in human ASD.

## Results

### Differential expression and phosphorylation in *Pten*^*m3m4*^ brain

**Fig. 1:**
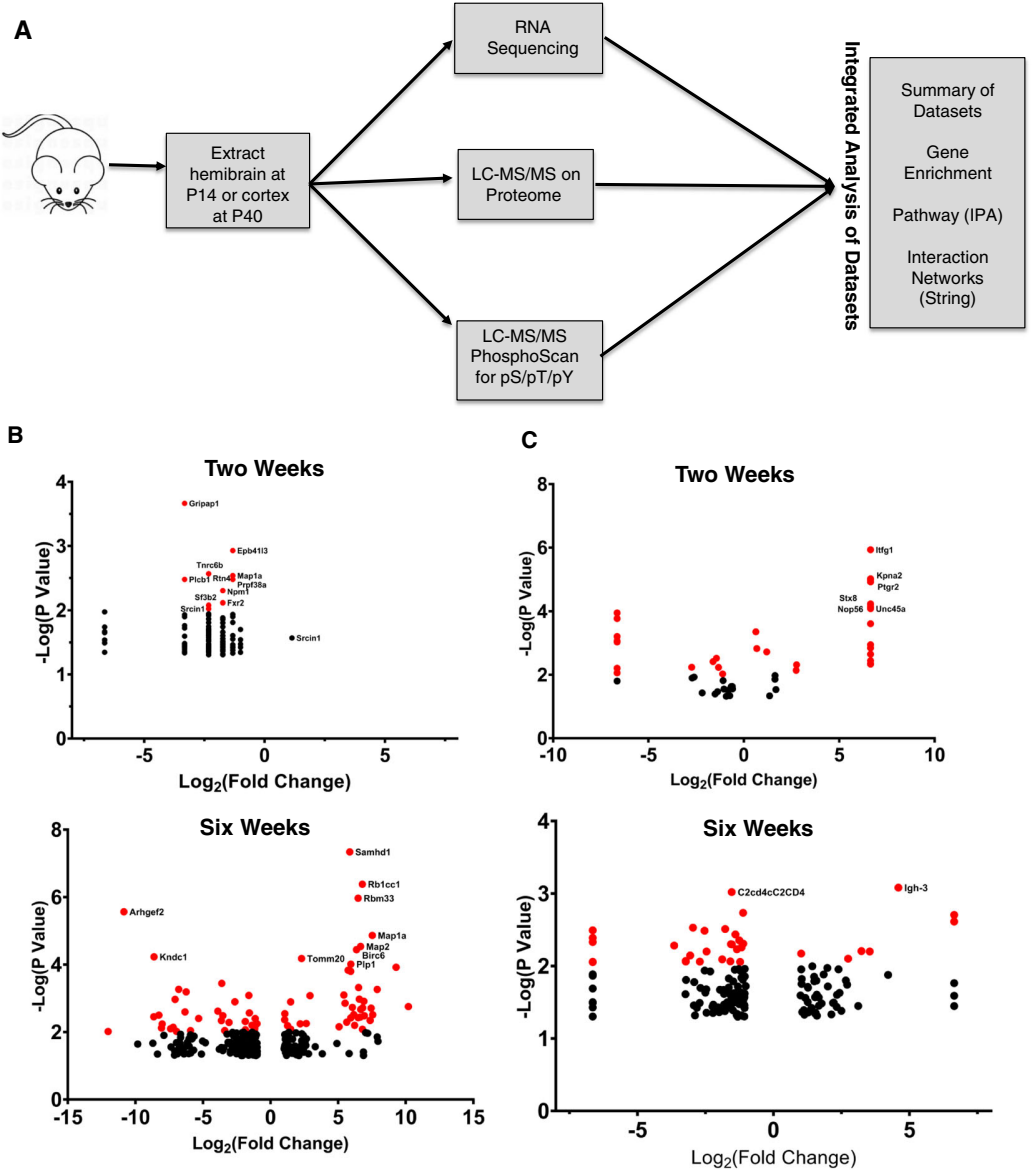
Omic profiling of *Pten*^*m3m4/m3m4*^ brain. **A** Hemibrain and cortical tissues were extracted from two-week-old (P14) and six-week-old (P40) *Pten*^*m3m4/m3m4*^ male mice (*N* = 3) for preparation for RNA sequencing, proteomic scanning, and pS/pT and pY phosphoproteomic scanning. After –omic profiling, these data were analyzed through various bioinformatics approaches. **B** Volcano plot of differentially expressed protein assessed at P14 (left) and P40 (right) by proteomic scan. **C** Volcano plot of differentially phosphorylated proteins assessed at P14 (left) and P40 (right) by phosphoproteomic scan.

In order to assess how the m3m4 mutation affects the landscape of gene and protein expression and protein phosphorylation, we performed parallel transcriptomic^26^, proteomic, and phosphoproteomic experiments on *Pten*^*m3m4/m3m4*^ hemibrains and cortices at two-weeks- (P14) and six-weeks-of-age (P40), respectively (Fig. 1A). At P14, a total of 3207 unique proteins, with an average of 2345 unique proteins per sample, were identified (Supplementary Data 1: Table 1), whereas at P40, a total of 6635 unique proteins, with an average of 5381 unique proteins per sample, were identified (Supplementary Data 1: Table 2). Principal component analysis (PCA) based on the LFQ/NSAF abundance values for a given protein, at both time points revealed completely separate clusters representing homozygous mutant and wild-type samples (Supplementary Information: Fig. 1A, B). At P14, a total of 4080 unique peptides were identified, 97% of which contained phosphorylated residues in the phospho-serine/threonine scan, an average of 2239 unique phosphopeptides per sample (Supplementary Data 1: Table 3). At P40, a total of 8468 unique peptides were identified, 90% of which contained phosphorylated residues, with an average of 5783 unique phosphopeptides per sample (Supplementary Data 1: Table 4). PCA, based on the LFQ abundance values for a given phosphopeptide, at both time points revealed non-overlapping clusters of homozygous mutant and wild-type samples (Supplementary Information: Fig. 1C, D). These findings illustrate robust sampling of the proteome and phosphoproteome, and the captured variability differentiated homozygous mutant and wild-type brain samples well.

In assessing differentially expressed proteins, we found 24 over-expressed and 27 under-expressed proteins in the P14 mutants compared to wildtype controls (Table 1; Fig. 1B). At P40, we identified 150 over-expressed and 102 under-expressed proteins in the mutant brains compared to the wild-type brains (Table 1; Fig. 1B). When assessing differential phosphorylation of phospho-serine/threonine phosphopeptides, we found 1 increased and 99 decreased in the P14 mutant hemibrain (Table 1; Fig. 1C). Moreover, we found 113 increased and 185 decreased at P40 in the mutant cortex (Table 1; Fig. 1C). In the special phospho-tyrosine-specific phospho-scan, we identified 39 and 29 relatively increased phosphopeptides at P14 and P40, respectively (Table 1). In the same phospho-tyrosine scan, we found 29 and 26 decreased phosphopeptides at P14 and P40, respectively (Table 1). Overall, the over-expression/under-expression of proteins or enrichment/depletion of phosphopeptides were distributed roughly equally except in the P14 phospho-serine/threonine scan, where phosphopeptide depletion was heavily favored in the mutant by 99-fold (Table 1). Moreover, there is a general increase in differential expression and phosphorylation as the *Pten*^*m3m4/m3m4*^ mice age, a trend not observed in the phospho-tyrosine scan (Table 1).

**Table 1:** Summary of differentially expressed/phosphorylated molecules identified in the –omic datasets describing the *Pten*^*m3m4*^ (MUT vs WT) brain.

	P14 Proteome	P40 Proteome	P14 pS/pT Scan	P40 pS/pT Scan	P14 pY Scan	P40 pY Scan	P14 Transcriptome	P40 Transcriptome
Up	24	49	1	113	31	29	74	832
Down	27	102	99	185	29	26	15	458
Total	51	151	100	298	60	55	89	1290
Total Unique Molecules	51	151	84	235	52	52	89	1290

### Dissimilarity across –omic datasets describing the *Pten*^*m3m4*^ brain

**Fig. 2:**
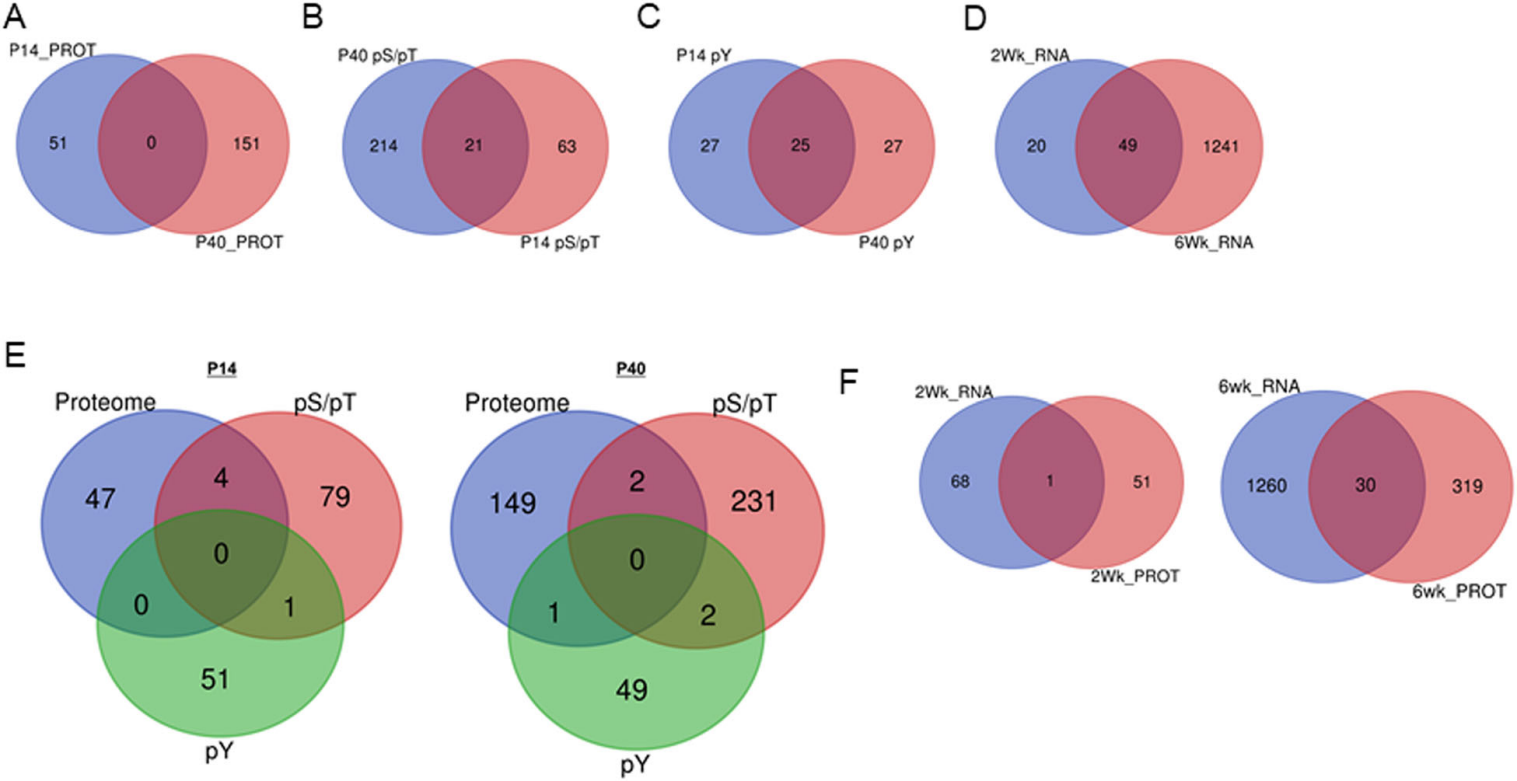
Minimal to marginal overlap among the various –omic datasets. **A** Venn diagram of protein IDs that are shared or not shared between P14 and P40 time points of the MUT vs WT proteomic scan. **B** Venn diagram of protein IDs that are shared or not shared between P14 and P40 time points of the MUT vs WT pS/pT phosphoproteomic scan. **C** Venn diagram of protein IDs that are shared or not shared between P14 and P40 time points of the MUT vs WT pY phosphoproteomic scan. **D** Venn diagram of gene IDs that are shared or not shared between P14 and P40 time points of the MUT vs WT transcriptome. **E** Venn diagram of protein IDs shared or not shared among the MUT versus WT proteome, pS/pT phosphoproteome, and pY phosphoproteome within time points P14 (left) and P40 (right). **F** Venn diagram of gene IDs shared or not shared between the transcriptome versus proteome within time points P14 (left) and P40 (right).

Given the eight different –omic datasets spanning two-time points, we sought to understand what molecules, showing differential expression/phosphorylation in the homozygous mutant brain, were shared among these datasets. Thus, we performed pairwise comparisons of the molecule lists of each separate –omics approach that compared *Pten*^*m3m4/m3m4*^ to *Pten*^*+/+*^ mice for both P14 and P40 time points. Surprisingly, we found no intersection between the P14 and P40 proteome (0% overlap; Fig. 2A; Supplementary Information: Table 1). For the phospho-serine/threonine scan, we found 21 molecules of all input molecules shared between time points (13% overlap; Fig. 2B; Supplementary Information: Table 1). For the phospho-tyrosine scan, we found 25 molecules of all input molecules shared between time points (48% overlap; Fig. 2C; Supplementary Information: Table 1). Furthermore, 49 molecules were shared between the P14 and P40 transcriptome datasets (7% overlap; Fig. 2D; Supplementary Information: Table 1). These analyses indicate little to marginal overlap within the various –omic scans over developmental time.

Additionally, we assessed overlap among the proteomic and phosphoproteomic datasets within their respective time points. Surprisingly, we found minimal intersection across these datasets; 8% overlap for P14 datasets and 3% overlap for P40 datasets (Fig. 2E; Supplementary Information: Table 1). We proceeded to assess the overlap between the transcriptome and proteome within each time point and found 1 (i.e., 2% overlap) and 30 (i.e., 4% overlap) intersecting molecules at P14 and P40, respectively (Fig. 2F; Supplementary Information: Table 1). Although we expected to find more shared genes between the transcriptome and proteome, these observations suggest that the mechanisms governing changes in protein expression are separate from those governing gene expression in our model. Moreover, the general lack of overlap among the various datasets suggests that in the context of *Pten* disruption, there may be little redundancy in modes of dysregulation, meaning that molecules are either subject to dysregulation via expression or phosphorylation but not both.

### Differentially expressed/phosphorylated molecules associate with major intracellular signaling cascades, neurological processes, and cancer

**Fig. 3:**
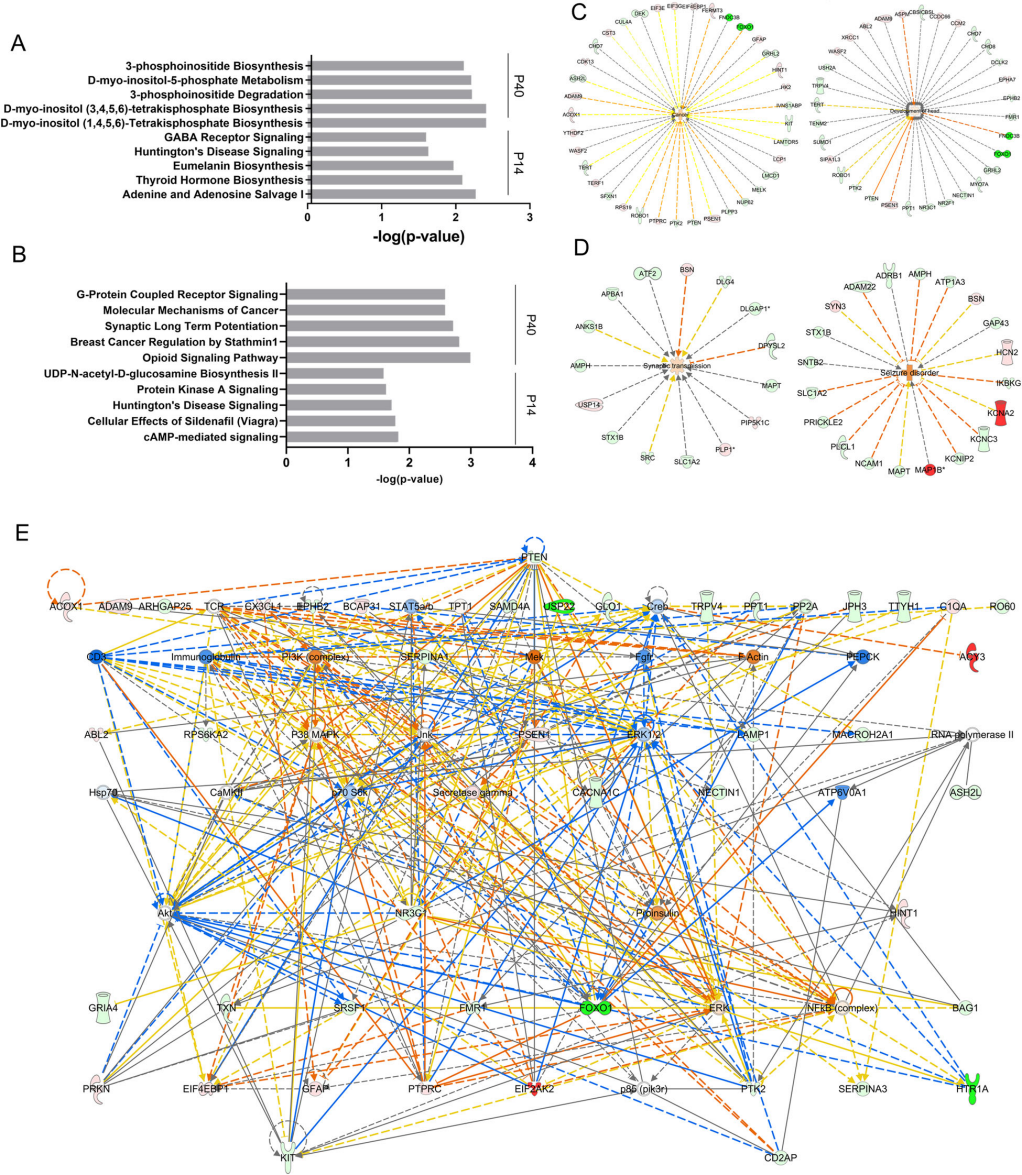
Evaluation of the biological impact of the proteomic and phosphoproteomic changes in the *Pten*^*m3m4/m3m4*^ brain. **A** The top five most significant “Canonical Pathways” identified by IPA core analysis in the P40 cortical and P14 hemibrain proteome comparing *Pten*^*m3m4/m3m4*^ mice to wildtype controls. **B** The top five most significant “Canonical Pathways” identified by IPA core analysis in the P40 cortical and P14 hemibrain phosphoproteome comparing *Pten*^*m3m4/m3m4*^ mice to wildtype controls. **C** The top two “disease and bio function” networks (by size) identified by IPA core analysis from the P40 cortical proteome of *Pten*^*m3m4/m3m4*^ mice compared to wildtype controls with activity prediction overlaid. Green = decreased expression. Red = increased expression. Color intensity = degree of increased/decreased expression. Hashed line = indirect biological relationship. Arrow = Direction of relationship. Orange = predicted activation. Yellow = observation inconsistent with direction of prediction. Gray = no influence on prediction. **D** The top two “disease and bio function” networks (by size) identified by IPA core analysis from the P40 cortical phosphoproteome of *Pten*^*m3m4/m3m4*^ mice compared to wildtype controls with activity prediction overlaid. Green = decreased phosphorylation. Red = increased phosphorylation. Color intensity = degree of increased/decreased phosphorylation. Hashed line = indirect biological relationship. Arrow = Direction of relationship. Orange = predicted activation. Yellow = observation inconsistent with direction of prediction. Gray = no influence on prediction. **E** The top network identified by IPA from among the differentially expressed proteins with Molecular Activity Predictor (MAP) results overlaid. The network is organized hierarchically, meaning the most important regulatory molecule is placed on top. Green = decreased expression. Red = increased expression. Color intensity = degree of increased/decreased expression. Solid line = direct biological relationship. Hashed line = indirect biological relationship. Arrow = direction of relationship. Orange = predicted activation. Blue = predicted inactivation. Yellow = observation inconsistent with the direction of prediction. Gray = no influence on prediction.

To understand the biological consequences of the observed changes in protein expression and phosphorylation in the *Pten*^*m3m4/m3m4*^ cortex, we performed an IPA analysis on the proteomic and phosphoproteomic (pooled phospho-serine/threonine and phospho-tyrosine datasets) findings. The comprehensive summaries for all IPA analyses can be found in the supplemental information (Supplementary Information). The top five most enriched canonical pathways at P14 and P40 for the proteome and phosphoproteome are shown in Fig. 3A, B, respectively. In the *Pten*^*m3m4/m3m4*^ proteome, the top implicated canonical pathways broadly involve phosphoinositide metabolism (i.e., 3′-phosphoinositide Biosynthesis) or neurological pathways, such as GABA Receptor Signaling and Glutamate Receptor Signaling (Fig. 3A; Supplementary Data 2: Tables S1, S2). In the *Pten*^*m3m4/m3m4*^ phosphoproteome, the top canonical pathways generally involve neurological and cancer processes or major cellular signaling cascades: Molecular Mechanisms of Cancer, Synaptic Long Term Potentiation, Neuregulin Signaling and cAMP-mediated Signaling (Fig. 3B; Supplementary Data 2: Tables 3, 4). Moreover, we extracted the top two salient disease and bio function networks (i.e., the networks comprised of the largest number of differentially expressed/phosphorylated molecules) from the proteome and phosphoproteome at P40. Cancer and Development of Head are the two largest disease and bio function networks identified by IPA in the *Pten*^*m3m4/m3m4*^ P40 cortical proteome (Fig. 3C; Supplementary Information), and Synaptic Transmission and Seizure Disorder are two of the largest salient networks from the *Pten*^*m3m4/m3m4*^ P40 cortical phosphoproteome (Fig. 3D; Supplementary Information). Overall, a broad qualitative appraisal of these enriched pathways and functional networks consistently implicate PTEN signaling, overgrowth or proliferative processes, and neurological process, especially those related to synaptic transmission or neuroinflammation (Supplemental Data 4). These enrichment analyses highlight the broad yet related biological perturbations in the neural (phospho)proteome of *Pten*^*m3m4/m3m4*^ mice.

In an effort to understand more about how these biological changes are initiated and maintained, we sought to identify important upstream regulators via network analysis of the differentially expressed proteins. Strikingly, the top protein interaction network identified by IPA from the *Pten*^*m3m4/m3m4*^ P40 cortical proteome agnostically positions Pten as the pre-eminent regulatory node via hierarchical ordering. The position of Pten at the top of this network’s regulatory architecture establishes its importance and influence as a regulator of the molecules positioned below (Fig. 3E). When IPA’s biological predictions are overlaid on the network using the Molecule Activity Predictor (MAP) tool, the observed decrease in Pten expression predicts an increase in the activity of Pi3k, Akt, Erk, p70 S6, Gfap, and C1qa, while predicting a decrease in P38 Mapk and Creb (Fig. 3E). These predictions are largely consistent with previous observations in the *Pten*^*m3m4/m3m4*^ cortex^22,24,25^. The network analysis enables a biological understanding of the changes in protein expression and phosphorylation, which unequivocally point to disrupted Pten function in the brain and highlight its importance in propagating dysfunction to downstream effector molecules.

### Pten and Psd-95 are major regulatory nodes in the *Pten*^*m3m4/m3m4*^ cortex

**Fig. 4:**
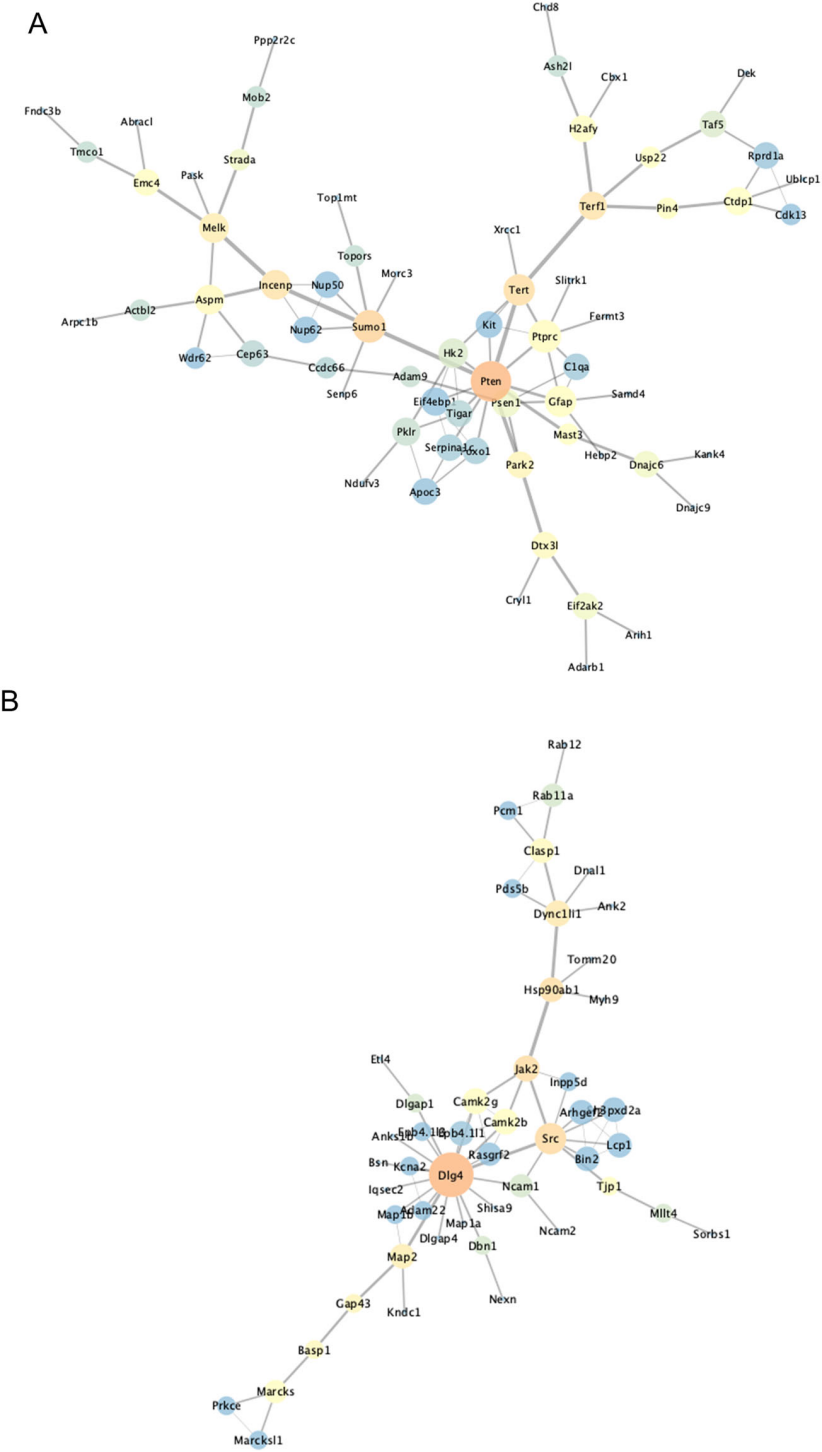
Network analysis identifies major regulatory nodes in –omic data, such as Pten and Psd-95 (i.e., Dlg4). **A** Largest network of related molecules constructed from P40 proteomic results (MUT vs WT) using STRING database. Node size trends with degree connectivity. Edge size trends with relationship evidence. Node color (warmth) trends with betweenness centrality. **B** Largest network of related molecules constructed from P40 pS/pT phosphoproteomic results (MUT vs WT) using STRING database. Node size trends with degree connectivity. Edge size trends with relationship evidence. Node color (warmth) trends with betweenness centrality.

In an effort to expand our biological understanding of the results from the proteomic and phosphoproteomic *Pten*^*m3m4/m3m4*^ versus *Pten*^*+/+*^ comparisons, we performed STRING analyses on each to identify the relationships among the differentially expressed/phosphorylated molecules. We extracted the largest STRING networks from each –omic dataset at each time points and analyzed the network statistics using Cytoscape (Supplementary Information: Fig. 2). The most striking network findings were observed in the P40 *Pten*^*m3m4/m3m4*^ cortical proteome and phosphoproteome. In the largest network (83 nodes) constructed from the P40 *Pten*^*m3m4/m3m4*^ cortical proteome findings, we found that Pten exhibited the greatest degree of connectivity, 12, and betweenness centrality, 0.66, relative to all other nodes (Fig. 4A). These data independently identify Pten as the most connected (i.e., degree connectivity) and most important node for transmitting information across the network (i.e., betweenness centrality), suggesting that Pten is likely to be the dominant regulatory node affecting the network of differentially expressed proteins in the P40 mutant cortex. Additionally, in the largest network (92 nodes) constructed from the P40 *Pten*^*m3m4/m3m4*^ cortical phospho-serine/threonine scan, we found Psd-95 (also known as Dlg4) exhibited the greatest degree connectivity, 19, and betweenness centrality, 0.68, relative to all other nodes (Fig. 4B). These data identify Psd-95 as the most connected (i.e., highest degree connectivity) and most important node for transmitting information across the network (i.e., highest betweenness centrality), suggesting that Psd-95 is likely to be the dominant regulatory node affecting the network of differentially phosphorylated proteins in the P40 mutant cortex. The Psd-95 finding also implicates Pten given the known and well-described protein-protein relationship between the two^30^. Ultimately, the STRING network analysis implicates Pten and Psd-95 as the likely perpetrators of the proteomic and phosphoproteomic dysregulation observed in the cortex of the *Pten*^*m3m4/m3m4*^ model and possibly responsible for some of the pathological cellular, physiological, and behavioral phenotypes.

### Meaningful overlap between differentially expressed or phosphorylated molecules and known ASD risk genes

**Fig. 5:**
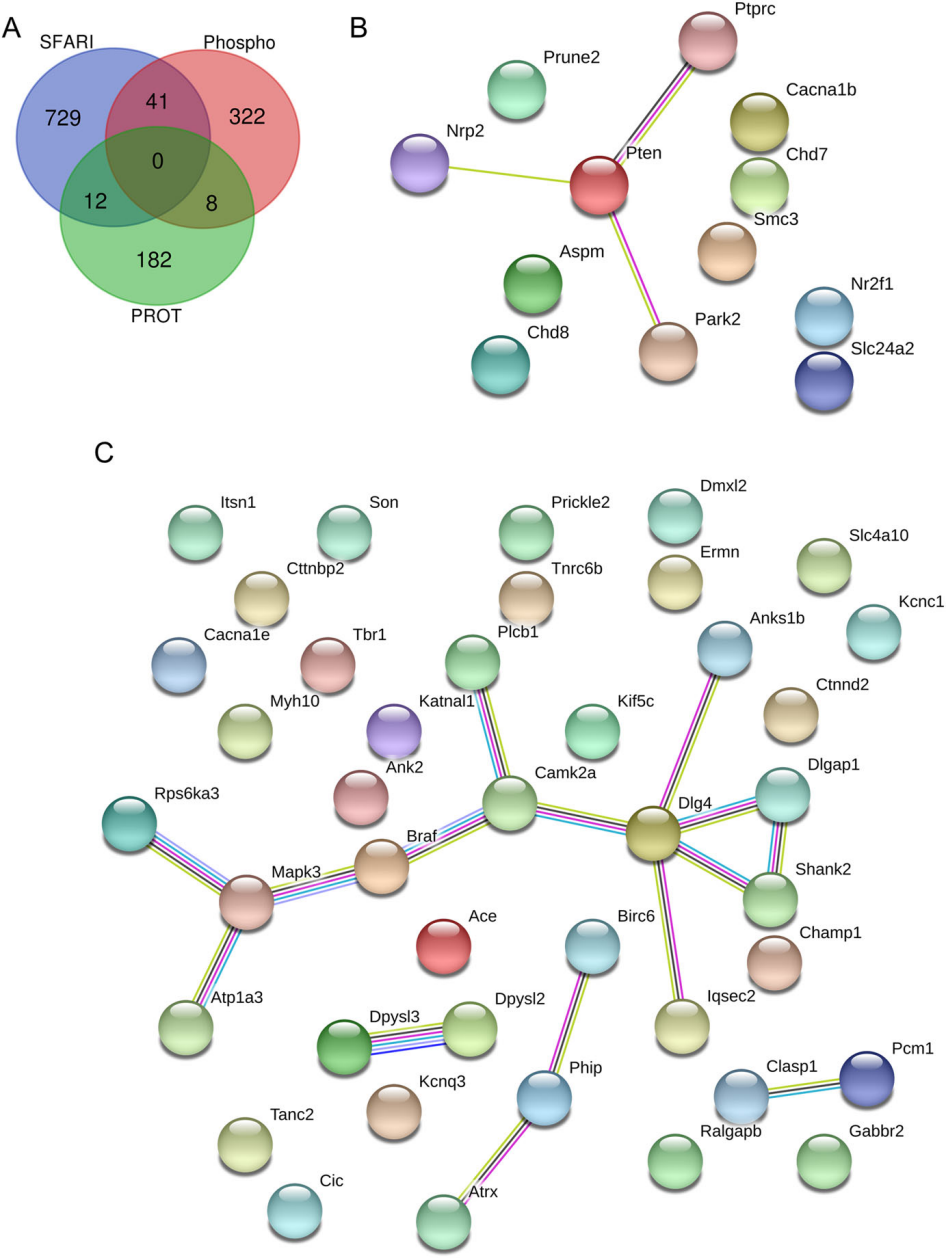
Differentially expressed and phosphorylated proteins in the *Pten*^*m3m4/m3m4*^ brain show overlap with known autism risk genes as curated by SFARI. **A** Venn diagram illustrating the number of shared and not shared targets among autism risk genes (SFARI), the *Pten*^*m3m4/m3m4*^ CNS proteome, and the *Pten*^*m3m4/m3m4*^ CNS phosphoproteome. **B** Proteins differentially expressed in the *Pten*^*m3m4/m3m4*^ CNS proteome that are known autism risk genes with biological relatedness overlaid via STRING database. **C** Proteins differentially phosphorylated in the *Pten*^*m3m4/m3m4*^ CNS phosphoproteome that are known autism risk genes with biological relatedness overlaid via STRING database.

We sought to assess whether the differentially expressed/phosphorylated molecules identified by our –omic surveys demonstrate significant overlap with known autism risk genes as curated by the Simons Foundation Autism Research Initiative (SFARI). Accordingly, we compared the SFARI catalog of ASD risk genes with the gene lists of the significant results of –omic surveys (time points and phospho-serine/threonine/tyrosine pooled). We found that 41 molecules intersected between the phosphoproteome and SFARI genes, and 12 molecules intersected between the proteome and SFARI (Fig. 5A). This was modest overlap given the number of genes curated by SFARI, but again, STRING network analysis implicated Pten and Psd-95 as central nodes in association networks derived from the intersecting molecules (Fig. 5B, C). The re-emergence of Pten and Psd-95 in a separate network analysis predicated on known ASD risk genes strengthens the evidence that implicates them as potential drivers of the phenotypes observed in *Pten*^*m3m4/m3m4*^ mouse model. The overlap that does exist with the known ASD risk genes underscores the importance of Pten biology to ASD pathophysiology overall.

## Discussion

This study represents a proteomic and phosphoproteomic exploration of the brain of a Pten model with strong neurological phenotypes. These experiments demonstrate a distinct developmental difference in protein expression and phosphorylation with differences of greater number, magnitude, and significance at P40 versus P14 (Fig. 1). Interestingly, we found little to marginal overlap among the various –omic datasets, but that the affected biological pathways across the different datasets were similar, if not overlapping (Figs. 2 and 3), suggesting potential convergence of non-overlapping, non-redundant regulatory mechanisms. Additionally, the developmental differences and lack of overlap may be a product of the significant decline in Pten expression from P14 to P40^24^. Network analysis highlighted Pten as a critical regulator of the differential expression observed in the neural proteome (Figs. 3E and 4A), and Psd-95 as a critical regulator of differential phosphorylation observed in the neural phosphoproteome (Fig. 4B). Ultimately, we observed overlap between our scans’ findings and known ASD risk genes, which when that overlap was subject to network analysis, we found Pten and Psd-95 to again be prominent nodes (Fig. 5).

The lack of overlap among the –omic datasets, especially the *Pten*^*m3m4/m3m4*^ neural proteome and transcriptome, is likely our most surprising finding. As this is the first exploration of a transcriptome and proteome simultaneously in a *Pten* model, it is difficult to comment whether this finding would be typical. Apparently in mice, the average correlation between the transcriptome and proteome is modest, 27%, which has been shown to be partially explained by alternative splicing^31^. Our findings still show less overlap than would be expected based on the aforementioned average. However, this overlap approaches expectations if the overlap analysis is repeated with a relaxed significance threshold (p-value < 0.10). Otherwise, these findings may indicate that gene versus protein expression, at least in our model, are regulated by different mechanisms, and perhaps reflecting a lack of redundancy. It is possible that dysregulated transcription and splicing factor expression may be responsible in part for changes in gene expression^26,27^, whereas translational mechanisms subsequent to changes in the phosphoproteome, including signaling changes downstream of Pten (e.g. dysregulation of translational control from changes in mTOR activity) may be responsible for the changes in protein expression. The possibility of significant changes in translational control in the *Pten*^*m3m4/m3m4*^ brain are real and tantalizing; disruption of proteins involved in translational control, including many in the PI3K/AKT/mTOR cascade, have been shown to mediate ASD phenotypes^32^. It is also possible that changes in alternative splicing may affect the differential protein expression that has been observed in the *Pten*^*m3m4/m3m4*^ brain^27^. Furthermore, the lack of overlap between the proteome and phosphoproteome suggests differential modes of regulation at these levels with minimal redundancy. Thus, mechanisms that regulate protein expression appear to be distinct from the mechanisms that regulate phosphorylation the *Pten*^*m3m4*^ brain, and a phenomenon previously observed and described in other models^33^. This differential regulation seems like a reasonable possibility in our model given that it is probable that a portion of the observed changes in the *Pten*^*m3m4/m3m4*^ neural phosphoproteome are more proximal to the dysfunction introduced by the m3m4 mutation than the differential gene/protein expression. This is likely due to the primary functional role of Pten as a dual-specificity phosphatase with possibly a large array of diverse protein substrates^34–42^. As mentioned above, the significant developmental decline in Pten expression may explain some of the differences observed between datasets. Moreover, the potential lack of redundancy across different –omic levels appears consistent with the autosomal dominant pattern of PHTS. Additional biochemical experiments and expanded –omic surveys on this model and other various neural Pten models will be required to disentangle the complexity at the various biological levels.

Beyond the lack of overlap between transcriptome and proteomes, we found the continued emergence of Pten and Psd-95 in the proteome and phosphoproteome pathway and network analysis to be a crucial indicator of their importance to the underlying pathology of the *Pten*^*m3m4/m3m4*^ brain and potentially to ASD in general. This postulate appears to be supported by one of the only two proteomic analyses of post-mortem ASD brains that identified Psd-95 as a major hub protein in the Brodmann area 19 (BA19)^19^. Although their finding was in the proteome, whereas ours was in the phosphoproteome, the shared finding is striking, and it is also possible that the effect of the perturbations to Psd-95 have similar biological effects in both contexts. There were other hits that our study shared with that of Abraham and colleagues (e.g. MAPT), suggesting proteomic approaches to ASD may uncover shared pathological hallmarks.

The picture of the broader changes in the (phospho)proteome of the *Pten*^*m3m4/m3m4*^ brain was largely aligned with our hypotheses, and in many ways, consistent with the overall pathophysiological changes we observed previously in the *Pten*^*m3m4/m3m4*^ neural transcriptome^26^. Moreover, many of the differentially expressed or phosphorylated proteins were previously identified in prior phenotyping efforts, including many molecules related to microglia and oligodendrocyte functions like C1q and Mbp^23,24^. Although many of the specific molecules and pathways found to be dysregulated or enriched, respectively, are different across the various datasets, there is a convergence on biological pathways downstream of Pten functions and disease processes that manifest in individuals with germline *PTEN* mutations, such as neurodevelopmental abnormalities and cancer (Supplementary Information, Supplementary Data 2). Interestingly, the co-occurrence of neurodevelopmental and cancer pathways arising from study of *Pten*^*m3m4/m3m4*^ brain underscores how closely the biology of these two seemingly disparate disease processes are and that an important nexus between them is PTEN. Our detailed description of the affected pathways and processes at the (phospho)proteomic levels appear to suggest that there may be convergent mechanisms at play in autism and that the apparent complexity might be reduced to a few central molecules and pathways despite a complex phenotype.

The *Pten*^*m3m4*^ model is excellent for the purposes of this study, but it is quite unique, and our study is limited to this model. Thus, our observations cannot necessarily be extended to all mutations or disruptions of *Pten* despite some of the evidence here and in the literature that support generalizability. In fact, it is quite likely that at least part of the expression and phosphorylation changes are specific to the m3m4 model due to the homozygosity of the mutation and cytoplasmic predominance of Pten expression–both features that are unique to our model and rarely, if ever, modeled in other studies. Additionally, Pten expression in the *Pten*^*m3m4*^ brain declines with age, likely through increased proteosomal degradation, and the m3m4 mutant protein is roughly 50% less stable than wildtype^24^. Our study would have ostensibly been more sensitive to changes in the proteome, phosphoproteome, and transcriptome with a larger sample size. Although the PCA findings cluster *Pten*^*m3m4/m3m4*^ and *Pten*^*+/+*^ separately, there is still enough variation within each genotype to indicate that our study would likely find more significant hits, if sample size expanded. Subsequently, we have likely only uncovered the most differentially expressed/phosphorylated molecules, but there may remain important subtle changes in major regulators that were missed.

In sum, our study represents an important entry into understanding the proteome and phosphoproteome in a *Pten* model with well described neurological pathologies and behavioral deficits. Given the current lack of knowledge of what the proteomic and phosphoproteomic landscapes should look like in the context PTEN-ASD or even idiopathic ASD, this study is a step toward filling this critical knowledge gap. The observed changes in protein expression and phosphorylation appear to affect pathways and processes important in the nervous system (e.g. neurodevelopment and synaptic function) and neuroinflammation. Despite the shared biological signature across the various –omic datasets, the implicated molecules appear to be distinct, which suggest differential modes of regulation across the different biological tiers. The molecules that do emerge from our study as major regulatory players (i.e. Pten and Psd-95) are strongly associated with autism biology. Our work suggests that a renewed focus on these molecules, specifically developing an understanding that would allow for rescue of their important functions in the nervous system, is critical if we are to tackle the clinical challenges presented by ASD.

## Methods

### The *Pten*^*m3m4*^ murine model

Our planned experimental procedures were approved by the Cleveland Clinic’s Institutional Animal Care and Use Committee (IACUC) under protocol numbers 2018-1952 and 2017-1879 and guided by the Principles of Laboratory Animal Care formulated by the National Society for Medical Research. We created the *Pten*^*m3m4*^ mouse on an outbred CD1 background, which has been studied extensively, shown to have deficits in social behavior, changes in neuron and glia populations, and changes in gene expression in many known autism risk genes^22–24,26,27,43^. The *Pten*^*m3m4*^ mutation, located within exon 7 of mouse *Pten*, consists of five nucleotide substitutions that results in four nonsynonymous and one synonymous codon changes. The four missense mutations disrupt the third (m3) and fourth (m4) putative nuclear localization-like sequences of Pten^21,22^. We performed mouse genotyping on genomic DNA extracted from toe clippings following Jackson Laboratory’s protocol using in-house PCR primers. The wildtype allele primers are mPten-F5, 5′-TGGCAGACTCTTCATTTCTGTGGC-3′, and mPten-R6, 5′-ACTTCTTCACAACCACTTCTTTCAAC-3′, and mutant allele primers are mPten-F3, 5′-TACCCGGTAGAATTTCGACGACCT-3′, and mPten-R6, 5′-ACTTCTTCACAACCACTTCTTTCAAC-3′. Our mice were maintained on a 14:10 light:dark cycle, where access to food and water was ad libitum. We maintained room temperature between 18 and 26 °C. Mice were euthanized via CO2 asphyxiation with additional cervical dislocation. None of the experiments were blinded but all were randomized and conducted under protocols approved by the Institutional Animal Care and Use Committee (IACUC) at Cleveland Clinic. In our experiments, we utilized only male mice because the *Pten*^*m3m4/m3m4*^ behavioral phenotypes are most pronounced in males, and the previous RNA-Seq experiment used only male mice^26,27^. A sample size of three biological replicates (*N* = 3) was used for each genotype and each time point of the study (i.e., P14 and P40). These time points were patterned after the original RNA-sequencing study^26^. Moreover, we wanted to capture a molecular neurodevelopment signature by using rough “pediatric” versus “adult” time points. The P14 time point represents this younger stage prior to weaning, while the P40 time point represents the oldest stage we can logistically capture (the mice have at least reached sexual maturity). The *Pten*^*m3m4/m3m4*^ mice exhibit premature mortality from suspected status epilepticus, rarely living beyond 6 weeks-of-age.

### Transcriptomic data analysis

The hemibrain and cortical transcriptome of two-week-old and six-week-old *Pten*^*m3m4*^ mice (GSE59318), respectively, shares a similar differential expression (DE) profile with that of idiopathic autism, as described in detail by Tilot et al.^26^. We deployed Ingenuity Pathway Analysis (IPA) software (Qiagen, Redwood City, California) to perform a core analysis on the DE genes from the hemibrain and cortex of *Pten* homozygous mutant and wild-type mice at both time points (i.e. P14 and P40). We also confirmed and extended this analysis with STRING software (version 11.0; https://string-db.org/). We performed STRING analysis on the same list of DE. These comparisons were of the homozygous mutant compared to the wildtype (i.e., MUT versus WT = MUT/WT Fold Change). The transcriptomic DE data are found in Supplementary Data 1.

### Proteomic scan

Three wildtype and three homozygous mutant two-week-old mouse hemibrains and three wildtype and three homozygous mutant six-week-old cortices were submitted to the Lerner Research Institute (LRI) Proteomics Core. These samples were homogenized in urea lysis buffer (20 mM HEPES, 9 M urea, 1X HALT phosphatase inhibitor, pH 8.0) with lysis Matrix D beads using FastPrep-24 from MP Biomedical. An aliquot from each tissue lysate was taken out for in-solution tryptic digestion. These samples were solubilized in 50 μL of in-solution digestion buffer, 6M urea, 100 mM Tris pH 8.0, reduced with DTT, alkylated with iodoacetamide, and then diluted to give a final urea concentration of less than 1 M for digestion. All samples were digested in-solution by adding 0.5 μg trypsin and incubating overnight at room temperature. A second aliquot of trypsin was added and digestion was continued for an additional 6 h. After digestion, the peptides were desalted and quantified using Thermo Scientific Pierce Quantitative Colorimetric Peptide Assay kit (Thermo Scientific #23275). A 100 μg peptide aliquot from each sample was dried down and reconstituted in 100 μL 20 mM ammonium formate (pH 10) for offline fractionation. Fractionation was performed by loading 90 μL onto an offline Hp-RP separation column (Waters, XBridge C18 3.5 μm, 2.1 × 100 mm, or Waters XBridge C18 3.5 μm, 2.1 × 150 mm) using an Agilent 1200 microflow pump. The samples were fractionated using a stepwise gradient at 250 μL/min for 40 min. A fraction collector was used for collection with a rate of 1 minute per fraction through the rest of the gradient. One of every four collections were combined and a total of 10 fractions per sample was obtained. Each combined fraction was lyophilized by vacuum centrifugation, and reconstituted in 30 μL 1% acetic acid for LC-MS/MS. The LC-MS system was a Finnigan LTQ-Obitrap Elite hybrid mass spectrometer system. The HPLC column was a Dionex 15 cm × 75 μm id Acclaim Pepmap C18, 2 μm, 100 Å reversed phase capillary chromatography column. Five μL volumes of the extract were injected and the peptides eluted from the column by an acetonitrile/0.1% formic acid gradient at a flow rate of 0.25 μL/min were introduced into the source of the mass spectrometer on-line. The microelectrospray ion source is operated at 2.5 kV. The digest was analyzed using the data dependent multitask capability of the instrument acquiring full scan mass spectra to determine peptide molecular weights and product ion spectra to determine amino acid sequence in successive instrument scans. The data were analyzed by using all CID spectra collected in the experiment to search the mouse UniProtKB databases with the search program Mascot and Sequest. These search results were then uploaded into the program Scaffold. The quantitation was performed by comparing the normalized spectral counts for these samples. The experimental comparison is between homozygous mutant and wildtype (i.e., MUT versus WT = MUT/WT Fold-Change). The proteomic screen data are found in Supplementary Data 1.

### Phospho-serine/threonine proteomic scan

Three wildtype and three homozygous mutant two-week-old mouse hemibrain and three wildtype and three homozygous mutant six-week-old cortices were submitted to the Lerner Research Institute (LRI) Proteomics Core. These samples were homogenized in urea lysis buffer (20 mM HEPES, 9M urea, 1X HALT phosphatase inhibitor, pH 8.0) with lysis Matrix D beads using FastPrep-24 from MP Biomedical (Solon, OH). The protein concentration was measured using a Pierce BCA assay kit. The proteins samples were reduced with DTT and alkylated with iodoacetamide. These proteins were then digested with trypsin overnight at room temperature. After digestion, the peptides were purified using Sep-Pak C18 spin columns (Waters Corporation, WAT 051910). Prior to enrichment, an 8 pmol aliquot of phosphopeptide standard mix was spiked into each sample. Phosphopeptide enrichment was performed using Pierce TiO2 Phosphopeptide Enrichment Spin Tips according to the manufacturer’s instructions. The enriched peptide samples were subjected to C18 clean-up prior to LC-MS/MS analysis. The LC-MS system was a ThermoScientific Fusion Lumos mass spectrometry system. The HPLC column was a Dionex 15 cm × 75 μm id Acclaim Pepmap C18, 2 μm, 100 Å reversed-phase capillary chromatography column. Five μL volumes of the extract were injected and the peptides eluted from the column by an acetonitrile/0.1% formic acid gradient at a flow rate of 0.25 μL/min were introduced into the source of the mass spectrometer online. The microelectrospray ion source is operated at 1.9 kV. The digest was analyzed using the data-dependent multitask capability of the instrument acquiring full scan mass spectra to determine peptide molecular weights and product ion spectra to determine amino acid sequence in successive instrument scans. The data were analyzed by using all CID spectra collected in the experiment to search the mouse UniProtKB databases with the programs Proteome Discoverer 2.2. The experimental comparison is between homozygous mutant and wildtype (i.e., MUT versus WT = MUT/WT Fold Change). The phosphoproteomic pS/pT screen data are found in Supplementary Data 1.

### Phospho-tyrosine proteomic scan

Three wildtype and three homozygous mutant hemibrain and cortical tissue samples from P14 and P40 mice were delivered to the Proteomics Services Group, Cell Signaling Technology (Danvers, MA). These samples were homogenized and subject to protease digestion yielding peptides. These extracted peptides were loaded directly onto a 50 cm × 100 μm PicoFrit capillary column packed with C18 reversed-phase resin. The column was developed with a 90-minute linear gradient of acetonitrile in 0.125% formic acid delivered at 280 nL/min. After C18 solid-phase extraction, the peptides were enriched for phospho-Y residue by immunoprecipitation using a phosphotyrosine pY-1000 motif antibody (CST #8954) on A/G beads. After immunoprecipitation the resin was washed, the phosphopeptides were eluted, and then analyzed by LC-MS/MS using Orbitrap-Fusion Lumos, ESI-HCD. The MS analysis was performed with the following parameters: MS Run Time 96 min, MS1 Scan Range (300.0–1500.00), Top 20 MS/MS (Min Signal 500, Isolation Width 2.0, Normalized Coll. Energy 35.0, Activation-Q 0.250, Activation Time 20.0, Lock Mass 371.101237, Charge State Rejection Enabled, Charge State 1+ Rejected, Dynamic Exclusion Enabled, Repeat Count 1, Repeat Duration 35.0, Exclusion List Size 500, Exclusion Duration 40.0, Exclusion Mass Width Relative to Mass, Exclusion Mass Width 10ppm). MS/MS spectra were evaluated using SEQUEST. Searches were performed against the most recent update of the Uniprot *Mus musculus* database with mass accuracy of +/−50 ppm for precursor ions and 0.02 Da for product ions. Results were filtered with mass accuracy of +/−5 ppm on precursor ions and the presence of the intended motif. The experimental comparison is between homozygous mutant and wildtype (i.e., MUT versus WT = MUT/WT Fold Change). The phosphoproteomic pY screen data are found in Supplementary Data 1.

### Bioinformatic analysis

In order to assess the biology impacted by the differential expression or differential phosphorylation observed in our various –omic datasets, we employed Ingenuity Pathway Analysis (IPA, www.qiagen.com/ingenuity) to perform core analysis, which provides enrichment scores as p-values for their various outputs (e.g. canonical pathways) determined by one-tailed Fisher’s exact test using Ingenuity Knowledge Base as a reference (restricted to only experimentally observed findings on the *mus musculus* background). Reported p-values were adjusted for multiple-testing using Benjamini-Hochberg (BH) procedure. We also used STRING software (https://string-db.org/) to obtain gene ontology, Reactome (https://reactome.org), and KEGG results. The STRING analysis served to both confirm the observation in IPA, which are based on a proprietary database, and to obtain the raw data to pass along to network analysis.

### Network analysis

We performed network analysis using Cytoscape 3.7.0 (https://cytoscape.org/) on the interaction data generated by STRING software (https://string-db.org/) on all of our –omic datasets. The STRING analysis generates an association network from an input gene list based on their database of biological relationships, which included several types of evidence: co-expression, text-mining, biochemical/genetic data, and previously curated pathway and protein-complex knowledge. Cytoscape 3.7.0 was utilized to generate the network figures and statistics, such as degree connectivity and betweenness centrality.

### Reporting summary

Further information on research design is available in the Nature Research Reporting Summary linked to this article.

## Supplementary information

Supplementary Information

Supplementary Data 1

Supplementary Data 2

Reporting Summary

## Data Availability

Any raw data or analyses related to this study are available from the corresponding author upon reasonable request. All proteomic and transcriptomic data are available in Supplementary Data 1. The transcriptomic data are also deposited in the Gene Expression Omnibus (GEO) database, accession code GSE59318. The (phospho)proteomic data are available via ProteomeXchange with identifier PXD025351.

## Code availability

All scripts related to this study are available from the corresponding author upon reasonable request.
