# Supplementary material for: Transcriptome-(phospho)proteome characterization of brain of a germline model of cytoplasmic-predominant Pten expression with autism-like phenotypes
Source: NPJ Genom Med. 2021 Jun 2;6:42. doi: 10.1038/s41525-021-00201-z (PMC8173008; doi:10.1038/s41525-021-00201-z)
Supplement: Supplementary file 1 — Supplementary Information [file 41525_2021_201_MOESM1_ESM.pdf]

Supplementary Information

A Dual-Omic Characterization of the Brain of a Constitutional Model of Cytoplasmic-Predominant Pten Expression with Autism-like Phenotypes

Stetson Thacker[1,2], Charis Eng[1,2,3,4,5,*]

[1]Genomic Medicine Institute, Lerner Research Institute, Cleveland Clinic, Cleveland, Ohio 44195, U.S.A.

[2] Department of Molecular Medicine, Cleveland Clinic Lerner College of Medicine, Case Western Reserve University, Cleveland, Ohio 44106, U.S.A.

[3] Center for Personalized Genetic Healthcare, Cleveland Clinic Community Care and Population Health, Cleveland, Ohio 44195, U.S.A.

[4] Department of Genetics and Genome Sciences, Case Western Reserve University School of Medicine, Cleveland, Ohio 44106, U.S.A.

[5] Germline High Risk Focus Group, CASE Comprehensive Cancer Center, Case Western Reserve University School of Medicine, Cleveland, OH 44106, U.S.A.

***Correspondence:** engc@ccf.org; Genomic Medicine Institute, Lerner Research Institute, Cleveland Clinic 9500 Euclid Avenue, Cleveland, OH 44195; Tel: (216) 444-3900

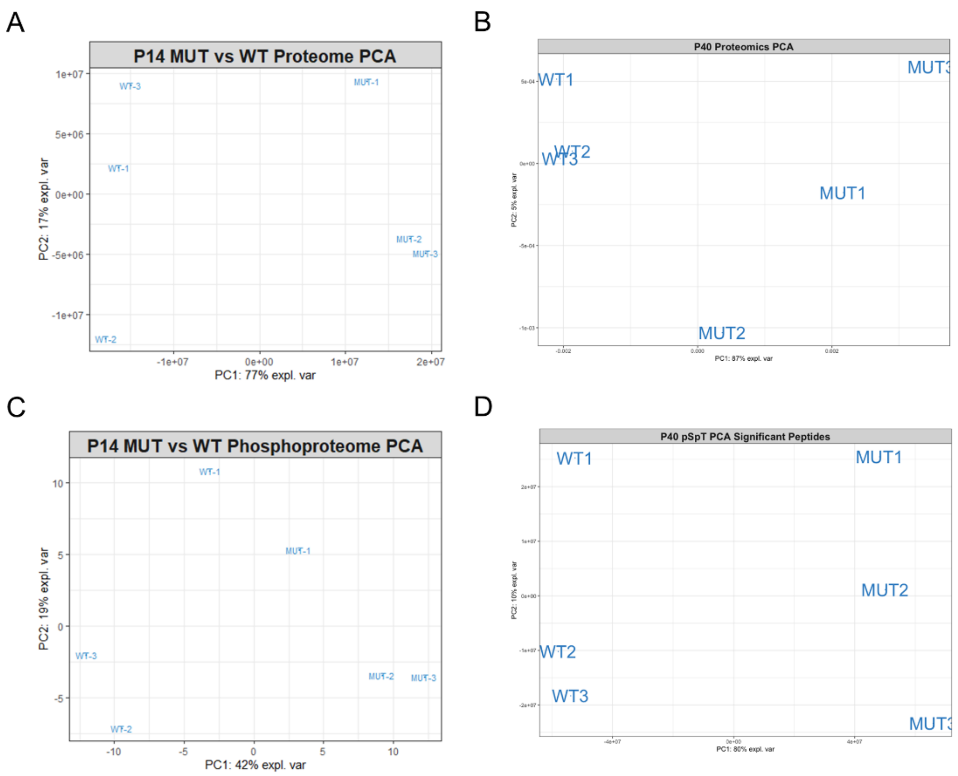

Fig. 1 Principal component analysis (PCA) on (phospho)proteomic data, showing complete separation between wildtype and mutant groups. **a** PCA of P14 proteome data. **b** PCA of P40 proteome data. **c** PCA of P14 pSpT phosphoproteome data. **d** PCA of P40 pSpT phosphoproteome data.

Table 1. Overlap analysis on –omic datasets between timepoints and approaches.

**Neural –omics comparisons**	**Overlap**
Proteome P14 vs P40 (Panel A)	0%
Phospho-S/T P14 vs P40 (Panel B)	13%
Phospho-Y P14 vs P40 (Panel C)	48%
Transcriptome P14 vs P40 (Panel D)	7%
Proteome vs Phospho-S/T/Y P14 (Panel E, Left)	8%
Proteome vs Phospho-S/T/Y P40 (Panel E, Right)	3%

Transcriptome vs Proteome P14 (Panel F, Left)	2%
Transcriptome vs Proteome P40 (Panel F, Right)	4%

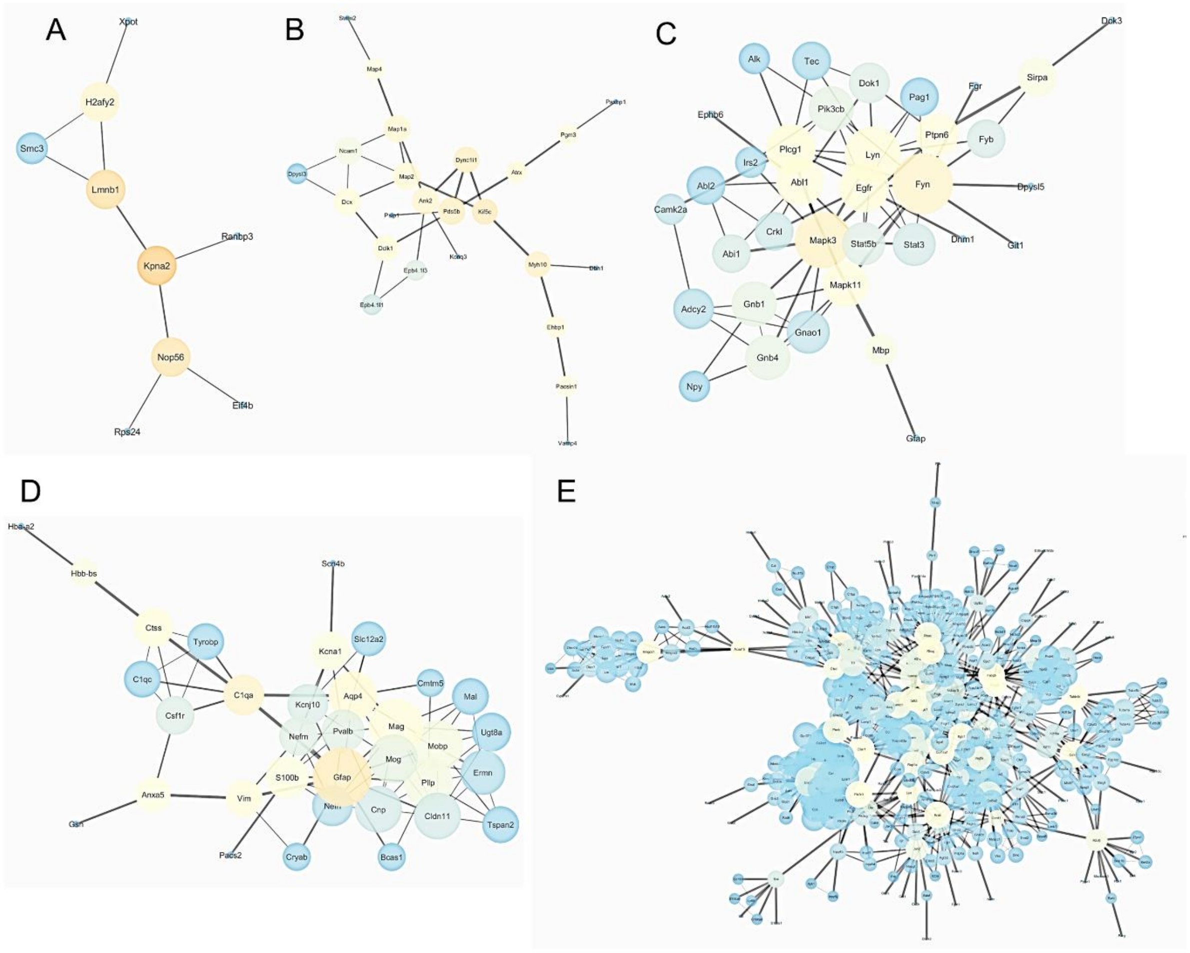

Fig. 2 Network analysis of the molecular interactions within each –omic datasets (excludes P40 pS/pT and proteomic networks shown in Fig. 4). **a** Largest network of related molecules constructed from P14 proteomic results (MUT vs WT) using STRING database. **b** Largest network of related molecules constructed from P14 pS/pT scan data (MUT vs WT) using STRING database. **c** Largest network of related molecules constructed from P14 and P40 pY scan data (MUT vs WT) using STRING database. **d** Largest network of related molecules constructed from P14 transcriptome data (MUT vs WT) using STRING database. **e** Largest network of related molecules constructed from P40 transcriptome data (MUT vs WT) using STRING database. For all panels: Node size trends with degree connectivity. Edge size trends with relationship evidence. Node color (warmth) trends with betweenness centrality.

INGENUITY
PATHWAY ANALYSIS

Analysis Name: Proteome_2wk_MUTvWT_IPA_Input - 2017-08-08 04:49 PM
Analysis Creation Date: 2017-08-08
Build version: 448560M
Content version: 36601845 (Release Date: 2017-06-22)

Analysis Settings

Reference set: Ingenuity Knowledge Base (Genes Only)

Relationship to include: Direct

Includes Endogenous Chemicals

Optional Analyses: My Pathways My List

Filter Summary:

Consider only relationships where

confidence = Experimentally Observed

(c) 2000-2017 QIAGEN. All rights reserved.

Top Canonical Pathways

Name	p-value	Overlap	
Adenine and Adenosine Salvage I	4.49E-03	50.0 %	1/2
Thyroid Hormone Biosynthesis	6.72E-03	33.3 %	1/3
Eumelanin Biosynthesis	1.12E-02	20.0 %	1/5
Huntington's Disease Signaling	1.73E-02	1.2 %	3/243
RhoA Signaling	3.17E-02	1.6 %	2/124

Top Upstream Regulators

Upstream Regulators

Upstream Regulator	p-value of overlap	Predicted Activation
HTT	1.04E-03	
RBBP5	6.84E-03	
ZNF282	9.11E-03	
MYC	1.08E-02	
TADA3	1.36E-02	

Causal Networks

Name	p-value of overlap	Predicted Activation
SOX4	3.44E-03	
YAF2	4.05E-03	
ELAVL1	5.75E-03	
CTNNB1	6.35E-03	
TIP60	7.48E-03	

Top Diseases and Bio Functions

(c) 2000-2017 QIAGEN. All rights reserved.

INGENUITY PATHWAY ANALYSIS

Diseases and Disorders

Name	p-value	#Molecules
Neurological Disease	2.88E-02 - 2.08E-05	23
Cancer	2.88E-02 - 4.38E-04	44
Gastrointestinal Disease	2.66E-02 - 4.38E-04	41
Organismal Injury and Abnormalities	2.88E-02 - 4.38E-04	47
Hereditary Disorder	1.78E-02 - 5.02E-04	15

Molecular and Cellular Functions

Name	p-value	#Molecules
Cellular Movement	2.88E-02 - 5.65E-05	16
Cell Death and Survival	2.66E-02 - 1.70E-04	22
Cellular Compromise	2.66E-02 - 2.68E-04	7
Cellular Assembly and Organization	2.88E-02 - 6.17E-04	14
Cellular Growth and Proliferation	2.67E-02 - 1.24E-03	17

Physiological System Development and Function

Name	p-value	#Molecules
Nervous System Development and Function	2.88E-02 - 2.38E-05	17
Organismal Development	2.88E-02 - 1.14E-03	18
Tissue Morphology	2.66E-02 - 1.45E-03	12
Auditory and Vestibular System Development and Function	2.25E-03 - 2.25E-03	1
Cardiovascular System Development and Function	2.66E-02 - 2.25E-03	6

Top Tox Functions

(c) 2000-2017 QIAGEN. All rights reserved.

INGENUITY PATHWAY ANALYSIS

Assays: Clinical Chemistry and Hematology

Name	p-value	#Molecules
Decreased Levels of Albumin	1.12E-02 - 1.12E-02	1
Increased Levels of Creatinine	3.10E-02 - 3.10E-02	1
Increased Levels of Hematocrit	1.95E-01 - 1.95E-01	1

Cardiotoxicity

Name	p-value	#Molecules
Pulmonary Hypertension	5.19E-03 - 5.19E-03	3
Heart Failure	1.86E-01 - 5.26E-02	2
Congenital Heart Anomaly	2.41E-01 - 7.58E-02	1
Cardiac Stenosis	1.06E-01 - 1.06E-01	1
Cardiac Dilation	1.44E-01 - 1.44E-01	2

Hepatotoxicity

Name	p-value	#Molecules
Liver Damage	3.78E-01 - 3.10E-02	2
Glutathione Depletion In Liver	4.40E-02 - 4.40E-02	1
Liver Fibrosis	1.13E-01 - 5.04E-02	2
Liver Necrosis/Cell Death	2.95E-01 - 1.45E-01	2
Liver Cirrhosis	1.79E-01 - 1.79E-01	2

Nephrotoxicity

Name	p-value	#Molecules
Renal Inflammation	1.83E-01 - 2.25E-03	3
Renal Nephritis	1.83E-01 - 2.25E-03	3
Glomerular Injury	2.25E-01 - 8.96E-03	4
Renal Fibrosis	1.18E-01 - 8.96E-03	1

(c) 2000-2017 QIAGEN. All rights reserved.

INGENUITY PATHWAY ANALYSIS

Renal Atrophy	4.40E-02 - 2.00E-02	1

Top Networks

ID	Associated Network Functions	Score
1	Cancer, Hematological Disease, Immunological Disease	27
2	Cancer, Organismal Injury and Abnormalities, Reproductive System Disease	25
3	Cellular Assembly and Organization, Neurological Disease, Hereditary Disorder	25
4	Cancer, Cellular Development, Cellular Growth and Proliferation	25

Top Analysis-Ready Molecules

Expr Log Ratio up-regulated

Molecules	Expr. Value	Expr. Chart
**ANKRD24**	↑ 10.000	
**CRYBB1**	↑ 10.000	
**GOLGA2**	↑ 10.000	
**ITFG1**	↑ 10.000	
**KPNA2**	↑ 10.000	
**METAP1**	↑ 10.000	
**MTA2**	↑ 10.000	
**NOP56**	↑ 10.000	
**PTGR2**	↑ 10.000	
**STX8**	↑ 10.000	

Expr Log Ratio down-regulated

Molecules	Expr. Value	Expr. Chart
**TSPAN2**	↓ -10.000	
**SMC3**	↓ -10.000	

(c) 2000-2017 QIAGEN. All rights reserved.

INGENUITY PATHWAY ANALYSIS

**RNF214**	↓ -10.000
**PRRT2**	↓ -10.000
**PIP4K2C**	↓ -10.000
**NRP2**	↓ -10.000
**GABRA1**	↓ -10.000
**ATRN**	↓ -10.000
**PTPN5**	↓ -6.655
**YES1**	↓ -6.548

(c) 2000-2017 QIAGEN. All rights reserved.

INGENUITY
PATHWAY ANALYSIS

Analysis Name: 6wks_M3M4_WTvMUT_Proteomics - 2020-02-03 02:53 PM
Analysis Creation Date: 2020-02-03
Build version: exported
Content version: 49932394 (Release Date: 2019-11-14)

Experiment Metadata

Name	Value
originalColumnNames	Accession

Analysis Settings

Reference set: Ingenuity Knowledge Base (Genes Only)
Relationship to include: Direct and Indirect
Includes Endogenous Chemicals
Optional Analyses: My Pathways My List

Filter Summary:
Consider only molecules and/or relationships where
(species = Mouse) AND
(confidence = Experimentally Observed) AND
(tissues/cell lines = Vd1 Gamma-delta T cells OR HL-60 OR Langerhans cells OR Cerebral Cortex OR Immune cell lines not otherwise specified OR Megakaryocytes OR Cerebral Ventricles OR Stem cells not otherwise specified OR Cervical cancer cell line not otherwise specified OR Pituitary Gland OR KM-12 OR Retina OR Other Lymphocytes OR Mononuclear leukocytes not otherwise specified OR Plasmacytoid dendritic

(c) 2000-2020 QIAGEN. All rights reserved.

cells OR Purkinje cells OR Vascular smooth muscle cells OR Microvascular endothelial cells OR U937 OR Intraepithelial T lymphocytes OR Tissues and Primary Cells not otherwise specified OR Kidney cell lines not otherwise specified OR BDCA-3+ dendritic cells OR COLO205 OR INS-1 OR LNCaP cells OR Other Immune cell lines OR Hep3B OR T47-D OR Myeloma Cell Lines not otherwise specified OR Other Macrophage Cancer Cell Lines OR MDA-MB-231 OR Osteosarcoma Cell Lines not otherwise specified OR U87MG OR Other Epithelial cells OR Smooth muscle cells not otherwise specified OR HeLa OR Prostate Cancer Cell Lines not otherwise specified OR PBMCs OR Other Cervical cancer cell line OR RXF-393 OR BT-474 OR UACC-257 OR Dermis OR Other Peripheral blood leukocytes OR Breast Cancer Cell Lines not otherwise specified OR Other Pancreatic Cancer Cell Lines OR Thyroid Gland OR Neurons not otherwise specified OR SR OR Other Nervous System OR Peripheral blood lymphocytes OR H460 OR Osteoblasts OR Other Bone marrow cells OR Other Lung Cancer Cell Lines OR Salivary Gland OR Endothelial cells not otherwise specified OR SNB-75 OR Effector memory cytotoxic T cells OR Activated Vd1 Gamma-delta T cells OR Microglia OR Activated Vd2 Gamma-delta T cells OR Choroid Plexus OR Effector T cells OR Mature monocyte-derived dendritic cells OR Peripheral blood monocytes OR Myeloid dendritic cells OR Other Macrophages OR Naive B cells OR Cardiomyocytes OR PANC-1 OR Teratocarcinoma Cell Lines not otherwise specified OR SK-MEL-28 OR SW-480 OR Spleen OR Pyramidal neurons OR Substantia Nigra OR Uterus OR CD56bright NK cells OR Parietal Lobe OR HCT-15 OR P19 OR UO-31 OR Epidermis OR Thymus OR A2780 OR CD56dim NK cells OR HMC-1 OR NCI-ADR-RES OR HEL OR Bladder OR Jurkat OR Other Prostate Cancer Cell Lines OR Other CNS Cell Lines OR NCI-H522 OR Other Cells OR Astrocytes OR Central memory helper T cells OR Smooth Muscle OR 293 cells OR Caco2 cells OR HuH7 OR Swiss 3T3 cells OR U266 OR Effector memory helper T cells OR Memory T lymphocytes not otherwise specified OR Hematopoietic progenitor cells OR HS 578T OR Chondrocytes OR Subventricular Zone OR MEF cells OR Other Hepatoma Cell Lines OR Amygdala OR Small Intestine OR HCT-116 OR Central memory cytotoxic T cells OR Pancreatic Cancer Cell Lines not otherwise specified OR Other Myeloma Cell Lines OR SF-295 OR Macrophage Cancer Cell Lines not otherwise specified OR Macrophages not otherwise specified OR Other Osteosarcoma Cell Lines OR MALME-3M OR MDA-MB-468 OR RAW 264.7 OR Sciatic Nerve OR Bone marrow-derived dendritic cells OR White Matter OR HOP-92 OR SK-OV-3 OR Lymph node OR BDCA-1+ dendritic cells OR HCC-2998 OR Other T lymphocytes OR NIH/3T3 cells OR Immature monocyte-derived dendritic cells OR Keratinocytes OR A498 OR CAKI-1 OR Pre-B lymphocytes OR Melanoma Cell Lines not otherwise specified OR Organ Systems not otherwise specified OR Activated CD56dim NK cells OR MDA-MB-435 OR Thymocytes OR Liver OR Mammary Gland OR Hepatocytes OR ACHN OR J774 OR Other Neuroblastoma Cell Lines OR Other B lymphocytes OR Hippocampus OR NK cells not otherwise specified OR THP-1 OR Other Breast Cancer Cell Lines OR Hypothalamus OR DU-145 OR Vd2 Gamma-delta T cells OR Leukemia Cell Lines not otherwise specified OR CNS Cell Lines not otherwise specified OR Adipocytes OR Eosinophils OR HT29 OR Embryonic stem cells OR Ovary OR HUVEC cells OR Other Tissues and Primary Cells OR Other Colon Cancer Cell Lines OR Lung OR Other Pheochromocytoma cell lines OR Stromal cells OR SF-268 OR M14 OR NCI-H226 OR WEHI-231 OR Splenocytes OR Beta islet cells OR Bone marrow cells not otherwise specified OR Lens OR Monocyte-derived dendritic cells not otherwise specified OR Nervous System not otherwise specified OR U251

(c) 2000-2020 QIAGEN. All rights reserved.

INGENUITY PATHWAY ANALYSIS

OR SN12C OR RPMI-8266 OR Caudate Nucleus OR Other Memory T lymphocytes OR Melanocytes OR Naive helper T cells OR Immune cells not otherwise specified OR OVCAR-8 OR Other Melanoma Cell Lines OR OVCAR-4 OR SK-MEL-2 OR Bone marrow-derived macrophages OR A375 OR Brainstem OR Granule cells OR Cos-7 cells OR Sertoli cells OR Nucleus Accumbens OR Olfactory Bulb OR Ventricular Zone OR RBL-2H3 OR LOX IMVI OR Spinal Cord OR Granulosa cells OR Other Cell Line OR Skeletal Muscle OR BT-549 OR Putamen OR Other Monocyte-derived dendritic cells OR SF-539 OR Trigeminal Ganglion OR Other Immune cells OR Other Teratocarcinoma Cell Lines OR MDA-N OR Skin OR Placenta OR MOLT-4 OR Pheochromocytoma cell lines not otherwise specified OR K-562 OR Kidney Cancer Cell Lines not otherwise specified OR Brain OR Large Intestine OR EKVX OR TK-10 OR Peripheral blood leukocytes not otherwise specified OR Ovarian Cancer Cell Lines not otherwise specified OR Murine NKT cells OR Pancreas OR Granule Cell Layer OR J-774A.1 OR Granulocytes not otherwise specified OR Th2 cells OR NCI-H23 OR Lung Cancer Cell Lines not otherwise specified OR Other Lymphoma Cell Lines OR Cytotoxic T cells OR SK-N-SH OR B lymphocytes not otherwise specified OR MG-63 OR SK-MEL-5 OR CD34+ cells OR Other Mononuclear leukocytes OR T lymphocytes not otherwise specified OR Mast cells OR Cornea OR Other Stem cells OR Medulla Oblongata OR IGROV1 OR Fibroblasts OR HOP-62 OR OVCAR-5 OR Other Leukemia Cell Lines OR Striatum OR Gray Matter OR 786-0 OR NT2/D1 OR OVCAR-3 OR Adrenal Gland OR Peritoneal macrophages OR Testis OR Min6 OR SW-620 OR Forestomach OR 3T3-L1 cells OR Heart OR Th1 cells OR Other Fibroblast cell lines OR Monocytes not otherwise specified OR Trachea OR Other Organ Systems OR Activated helper T cells OR Other Monocytes OR Cell Line not otherwise specified OR Blood platelets OR Corpus Callosum OR Other Dendritic cells OR Plasma cells OR Other Ovarian Cancer Cell Lines OR Monocyte-derived macrophage OR Prostate Gland OR Crypt OR Cortical neurons OR Other Smooth muscle cells OR Pro-B lymphocytes OR RKO OR Cells not otherwise specified OR Mesenchymal stem cells OR Other NK cells OR Dorsal Root Ganglion OR Adipose OR Other Kidney cell lines OR Cartilage Tissue OR CD4+ T-lymphocytes OR Dendritic cells not otherwise specified OR PC-3 OR Natural T-regulatory cells OR U2OS OR Memory B cells OR MCF7 OR Thalamus OR MDA-MB-361 OR Stomach OR Effector memory RA+ cytotoxic T cells OR HepG2 OR Colon Cancer Cell Lines not otherwise specified OR Other Granulocytes OR Esophagus OR Oocytes OR UACC-62 OR Activated CD56bright NK cells OR Other Endothelial cells OR NB4 OR Lymphocytes not otherwise specified OR Hepatoma Cell Lines not otherwise specified OR Cerebellum OR A549-ATCC OR Epithelial cells not otherwise specified OR PC-12 cells OR Other Neurons OR Lymphoma Cell Lines not otherwise specified OR Other Kidney Cancer Cell Lines OR Fibroblast cell lines not otherwise specified OR CCRF-CEM OR Th17 cells OR Neuroblastoma Cell Lines not otherwise specified OR Calvaria OR NCI-H332M OR Kidney OR Neutrophils OR BA/F3)
AND
(mol. types = biologic drug OR canonical pathway OR chemical - endogenous mammalian OR chemical - endogenous non-mammalian OR chemical - kinase inhibitor OR chemical - other OR chemical - protease inhibitor OR chemical drug OR chemical reagent OR chemical toxicant OR complex OR cytokine OR disease OR enzyme OR function OR G-protein coupled receptor OR group OR growth factor OR ion channel OR kinase OR ligand-dependent nuclear receptor OR mature microRNA OR microRNA OR other OR peptidase OR phosphatase OR transcription

(c) 2000-2020 QIAGEN. All rights reserved.

regulator OR translation regulator OR transmembrane receptor OR transporter) AND (data sources = An Open Access Database of Genome-wide Association Results OR BIND OR BioGRID OR Catalogue Of Somatic Mutations In Cancer (COSMIC) OR Chemical Carcinogenesis Research Information System (CCRIS) OR ClinicalTrials.gov OR ClinVar OR Cognia OR DIP OR DrugBank OR Gene Ontology (GO) OR GVK Biosciences OR Hazardous Substances Data Bank (HSDB) OR HumanCyc OR Ingenuity Expert Findings OR Ingenuity ExpertAssist Findings OR IntAct OR Interactome studies OR MIPS OR miRBase OR miRecords OR Mouse Genome Database (MGD) OR Obesity Gene Map Database OR Online Mendelian Inheritance in Man (OMIM) OR TarBase OR TargetScan Human)

Top Canonical Pathways

Name	p-value	Overlap
**D-myo-inositol (1,4,5,6)-Tetrakisphosphate Biosynthesis**	3.91E-03	3.8 % 5/133
**D-myo-inositol (3,4,5,6)-tetrakisphosphate Biosynthesis**	3.91E-03	3.8 % 5/133
**3-phosphoinositide Degradation**	5.96E-03	3.4 % 5/147
**D-myo-inositol-5-phosphate Metabolism**	6.13E-03	3.4 % 5/148
**3-phosphoinositide Biosynthesis**	7.82E-03	3.2 % 5/157

Top Upstream Regulators

Upstream Regulators

Name	p-value	Predicted Activation
**INS**	2.16E-03	

(c) 2000-2020 QIAGEN. All rights reserved.

INGENUITY PATHWAY ANALYSIS

OTUD3	7.92E-03
A830082K12Rik	7.92E-03
CLEC16A	7.92E-03
MIGA2	7.92E-03

Causal Network

Name	p-value	Predicted Activation
FGFR3	2.69E-03	
CDH1	6.96E-03	
OTUD3	7.92E-03	
A830082K12Rik	7.92E-03	
CLEC16A	7.92E-03	

Top Diseases and Bio Functions

Diseases and Disorders

Name	p-value range	# Molecules
Endocrine System Disorders	2.58E-02 - 1.86E-04	14
Metabolic Disease	2.80E-02 - 1.86E-04	14
Organismal Injury and Abnormalities	2.88E-02 - 1.86E-04	43
Infectious Diseases	2.32E-02 - 4.82E-04	6
Neurological Disease	2.88E-02 - 5.96E-04	18

(c) 2000-2020 QIAGEN. All rights reserved.

Molecular and Cellular Functions

Name	p-value range	# Molecules
Cell-To-Cell Signaling and Interaction	2.72E-02 - 9.73E-05	21
Cellular Development	2.73E-02 - 3.59E-04	24
Cellular Growth and Proliferation	2.32E-02 - 3.59E-04	23
Cellular Movement	2.58E-02 - 3.79E-04	14
Cell Morphology	2.32E-02 - 5.59E-04	21

Physiological System Development and Function

Name	p-value range	# Molecules
Nervous System Development and Function	2.88E-02 - 9.73E-05	28
Organ Development	2.32E-02 - 3.59E-04	13
Reproductive System Development and Function	2.32E-02 - 3.59E-04	5
Hematological System Development and Function	2.73E-02 - 3.79E-04	11
Hematopoiesis	2.73E-02 - 7.36E-04	7

Top Tox Functions

Assays: Clinical Chemistry and Hematology

(c) 2000-2020 QIAGEN. All rights reserved.

INGENUITY PATHWAY ANALYSIS

Name	p-value range	# Molecules
**Increased Levels of ALT**	1.72E-01 - 1.72E-01	1
**Increased Levels of Hematocrit**	5.37E-01 - 5.37E-01	1

Cardiotoxicity

Name	p-value range	# Molecules
**Cardiac Enlargement**	1.53E-01 - 3.16E-02	5
**Cardiac Proliferation**	7.76E-02 - 7.76E-02	2
**Congenital Heart Anomaly**	5.07E-01 - 2.22E-01	2
**Cardiac Necrosis/Cell Death**	2.49E-01 - 2.49E-01	2
**Heart Failure**	2.78E-01 - 2.78E-01	2

Hepatotoxicity

Name	p-value range	# Molecules
**Liver Hyperplasia/Hyperproliferation**	5.58E-01 - 2.32E-02	4
**Biliary Hyperplasia**	3.84E-02 - 3.84E-02	1
**Glutathione Depletion In Liver**	1.04E-01 - 4.59E-02	2
**Liver Fibrosis**	4.59E-02 - 4.59E-02	1
**Liver Damage**	2.26E-01 - 6.08E-02	3

Nephrotoxicity

(c) 2000-2020 QIAGEN. All rights reserved.

INGENUITY PATHWAY ANALYSIS

Name	p-value range	# Molecules
**Glomerular Injury**	1.52E-01 - 6.81E-02	3
**Renal Thrombosis**	6.81E-02 - 6.81E-02	1
**Renal Inflammation**	1.52E-01 - 8.60E-02	3
**Renal Nephritis**	1.52E-01 - 8.60E-02	3
**Renal Necrosis/Cell Death**	2.40E-01 - 2.40E-01	1

Top Regulator Effect Networks

Top Networks

ID	Associated Network Functions	Score
**1**	Cell Signaling, Cellular Function and Maintenance, Molecular Transport	36
**2**	Cancer, Organismal Injury and Abnormalities, Cell Death and Survival	17
**3**	Carbohydrate Metabolism, Molecular Transport, Small Molecule Biochemistry	17
**4**	Cell Cycle, Organismal Development, Reproductive System Development and Function	17

(c) 2000-2020 QIAGEN. All rights reserved.

INGENUITY PATHWAY ANALYSIS

5	Cancer, Organismal Injury and Abnormalities, Cell Death and Survival	17

Top Tox Lists

Top My Lists

Top My Pathways

Top Analysis-Ready Molecules

Expr Fold Change

Molecules	Expr. Value	Chart
ACY3	↑ 100.000	
EIF2AK2	↑ 100.000	
HEBP2	↑ 100.000	
HPF1	↑ 100.000	
MAN1B1	↑ 100.000	
Ighg2b	↑ 24.101	
TTLL7	↑ 18.540	
ARPC1B	↑ 11.620	
YTHDF2	↑ 9.401	

(c) 2000-2020 QIAGEN. All rights reserved.

INGENUITY PATHWAY ANALYSIS

**DNAJC9**	↑ 8.656

Expr Fold Change

Molecules	Expr. Value	Chart
**USP22**	↓ -100.000	
**TOP1MT**	↓ -100.000	
**MOB2**	↓ -100.000	
**KIF16B**	↓ -100.000	
**HTR1A**	↓ -100.000	
**GAREM1**	↓ -100.000	
**FOXO1**	↓ -100.000	
**FNDC3B**	↓ -100.000	
**DTX3L**	↓ -100.000	
**DHTKD1**	↓ -100.000	

(c) 2000-2020 QIAGEN. All rights reserved.

INGENUITY PATHWAY ANALYSIS

Analysis Name: Phospho ID Adjusted Input - 2017-04-03 01:44 PM
Analysis Creation Date: 2017-04-03
Build version: 448560M
Content version: 33559992 (Release Date: 2017-03-28)

Analysis Settings

Reference set: Ingenuity Knowledge Base (Genes Only)

Relationship to include: Direct and Indirect

Includes Endogenous Chemicals

Optional Analyses: My Pathways My List

Filter Summary:

Consider only relationships where

confidence = Experimentally Observed

(c) 2000-2017 QIAGEN. All rights reserved.

Top Canonical Pathways

Name	p-value	Overlap
cAMP-mediated signaling	1.05E-02	1.8 % 4/223
Cellular Effects of Sildenafil (Viagra)	1.35E-02	2.3 % 3/130
Huntington's Disease Signaling	1.36E-02	1.7 % 4/241
Protein Kinase A Signaling	1.70E-02	1.3 % 5/392
G-Protein Coupled Receptor Signaling	2.03E-02	1.5 % 4/272

Top Upstream Regulators

Upstream Regulators

Upstream Regulator	p-value of overlap	Predicted Activation
MARK4	7.12E-04	
CSNK2A1	1.18E-03	Inhibited
PRKCG	5.04E-03	
phytic acid	1.11E-02	
KNDC1	1.11E-02	

Causal Networks

Name	p-value of overlap	Predicted Activation
diosgenin	4.94E-05	Activated
CK1/2	2.87E-04	Inhibited
MARK4	7.12E-04	
CSNK2A1	1.14E-03	Inhibited
MFGE8	3.67E-03	Inhibited

Top Diseases and Bio Functions

(c) 2000-2017 QIAGEN. All rights reserved.

INGENUITY PATHWAY ANALYSIS

Diseases and Disorders

Name	p-value	#Molecules
Neurological Disease	1.14E-02 - 1.57E-09	46
Cancer	1.14E-02 - 2.69E-08	75
Gastrointestinal Disease	7.61E-03 - 2.69E-08	70
Organismal Injury and Abnormalities	1.14E-02 - 2.69E-08	77
Ophthalmic Disease	1.14E-02 - 1.61E-05	10

Molecular and Cellular Functions

Name	p-value	#Molecules
Cellular Assembly and Organization	1.14E-02 - 2.49E-12	42
Cellular Function and Maintenance	1.14E-02 - 2.49E-12	34
Cell Morphology	1.14E-02 - 2.86E-11	37
Cellular Development	1.14E-02 - 1.85E-09	24
Cellular Growth and Proliferation	7.61E-03 - 1.85E-09	24

Physiological System Development and Function

Name	p-value	#Molecules
Nervous System Development and Function	1.14E-02 - 6.47E-12	41
Tissue Morphology	1.14E-02 - 2.86E-11	26
Organismal Development	1.14E-02 - 1.85E-09	38
Tissue Development	1.14E-02 - 1.85E-09	32
Organ Morphology	1.14E-02 - 6.10E-08	27

Top Tox Functions

(c) 2000-2017 QIAGEN. All rights reserved.

Assays: Clinical Chemistry and Hematology

Name	p-value	#Molecules
Decreased Levels of Potassium	9.80E-02 - 9.80E-02	1

Cardiotoxicity

Name	p-value	#Molecules
Cardiac Enlargement	2.67E-02 - 3.75E-03	5
Cardiac Dilation	3.10E-01 - 3.81E-03	3
Congenital Heart Anomaly	8.06E-02 - 4.07E-03	2
Cardiac Dysfunction	1.25E-01 - 1.25E-01	1
Cardiac Necrosis/Cell Death	1.25E-01 - 1.25E-01	1

Hepatotoxicity

Name	p-value	#Molecules
Liver Hyperplasia/Hyperproliferation	2.73E-01 - 2.38E-04	40
Hepatocellular Carcinoma	2.73E-01 - 3.75E-02	5
Liver Fibrosis	4.61E-01 - 8.41E-02	2
Liver Damage	3.81E-01 - 3.81E-01	1
Liver Inflammation/Hepatitis	5.13E-01 - 3.81E-01	2

Nephrotoxicity

Name	p-value	#Molecules
Renal Inflammation	7.61E-03 - 7.61E-03	1
Renal Nephritis	7.61E-03 - 7.61E-03	1
Renal Necrosis/Cell Death	3.10E-01 - 1.84E-01	3

(c) 2000-2017 QIAGEN. All rights reserved.

Top Networks

ID	Associated Network Functions	Score
1	DNA Replication, Recombination, and Repair, RNA Post-Transcriptional Modification, Psychological Disorders	51
2	Nervous System Development and Function, Neurological Disease, Cell Morphology	48
3	Developmental Disorder, Neurological Disease, Post-Translational Modification	27
4	Cell-To-Cell Signaling and Interaction, Inflammatory Disease, Inflammatory Response	24
5	Nucleic Acid Metabolism, Carbohydrate Metabolism, Small Molecule Biochemistry	18

Top Analysis-Ready Molecules

Phospho Fold Change down-regulated

Molecules	Expr. Value	Expr. Chart
**PALM2***	↓ -20.000	
**NSRP1**	↓ -20.000	
**MAP2**	↓ -20.000	
**KIF5C***	↓ -20.000	
**HDAC5**	↓ -20.000	
**GABBR2**	↓ -20.000	
**BRAF**	↓ -20.000	
**AMER2***	↓ -20.000	
**XPO6**	↓ -10.000	
**TNKS1BP1**	↓ -10.000	

(c) 2000-2017 QIAGEN. All rights reserved.

INGENUITY PATHWAY ANALYSIS

INGENUITY
PATHWAY ANALYSIS

Analysis Name: pSpTScan_6wk_Input_IPA - 2018-10-29 07:23 PM
Analysis Creation Date: 2018-10-29
Build version: 484108M
Content version: 45868156 (Release Date: 2018-10-09)

Experiment Metadata

Name	Value

Analysis Settings

Top Canonical Pathways

Name	p-value	Overlap	
**Opioid Signaling Pathway**	1.03E-03	3.7 %	9/246
**Breast Cancer Regulation by Stathmin1**	1.56E-03	3.8 %	8/211
**Synaptic Long Term Potentiation**	1.93E-03	4.8 %	6/126
**Molecular Mechanisms of Cancer**	2.61E-03	2.8 %	11/394
**G-Protein Coupled Receptor Signaling**	2.63E-03	3.2 %	9/282

(c) 2000-2018 QIAGEN. All rights reserved.

Top Upstream Regulators

Upstream Regulators

Name	p-value	Predicted Activation
**CDK5R1**	1.13E-05	
**TNIK**	2.47E-04	
**CDK5**	3.14E-04	
**NPC1**	4.84E-04	
**DAB1**	4.84E-04	

Causal Network

Name	p-value	Predicted Activation
**CDK5R1**	5.85E-06	
**CDK5R2**	1.06E-04	
**Laminin1**	2.01E-04	
**POU1F1**	2.18E-04	
**DAB1**	2.47E-04	

Top Diseases and Bio Functions

Diseases and Disorders

(c) 2000-2018 QIAGEN. All rights reserved.

INGENUITY PATHWAY ANALYSIS

Name	p-value range	# Molecules
**Dermatological Diseases and Conditions**	2.62E-04 - 5.01E-12	149
**Organismal Injury and Abnormalities**	5.47E-03 - 5.01E-12	213
**Cancer**	3.66E-03 - 6.76E-12	208
**Gastrointestinal Disease**	2.21E-03 - 3.56E-10	197
**Reproductive System Disease**	6.04E-04 - 5.55E-10	149

Molecular and Cellular Functions

Name	p-value range	# Molecules
**Cellular Assembly and Organization**	6.43E-03 - 6.31E-20	102
**Cellular Function and Maintenance**	6.43E-03 - 6.31E-20	108
**Cell Morphology**	6.43E-03 - 6.04E-16	90
**Cellular Development**	5.42E-03 - 6.04E-16	60
**Cellular Growth and Proliferation**	5.42E-03 - 6.04E-16	52

Physiological System Development and Function

Name	p-value range	# Molecules
**Nervous System Development and Function**	6.43E-03 - 6.04E-16	98
**Organismal Development**	4.46E-03 - 6.04E-16	78
**Tissue Development**	5.47E-03 - 6.04E-16	70
**Embryonic Development**	4.46E-03 - 5.84E-11	40
**Tissue Morphology**	6.43E-03 - 2.11E-10	66

(c) 2000-2018 QIAGEN. All rights reserved.

INGENUITY PATHWAY ANALYSIS

Top Tox Functions

Assays: Clinical Chemistry and Hematology

Name	p-value range	# Molecules
Increased Levels of Hematocrit	7.80E-02 - 7.80E-02	3
Increased Levels of Creatinine	1.34E-01 - 1.34E-01	1
Increased Levels of AST	1.69E-01 - 1.69E-01	1
Increased Levels of ALT	2.43E-01 - 2.43E-01	1
Decreased Levels of Potassium	2.51E-01 - 2.51E-01	1

Cardiotoxicity

Name	p-value range	# Molecules
Heart Failure	3.37E-01 - 3.12E-04	5
Cardiac Arrythmia	3.97E-01 - 1.03E-02	7
Cardiac Dilation	5.20E-01 - 1.03E-02	8
Cardiac Dysfunction	2.59E-01 - 1.03E-02	6
Pulmonary Hypertension	2.32E-01 - 1.03E-02	3

Hepatotoxicity

(c) 2000-2018 QIAGEN. All rights reserved.

INGENUITY PATHWAY ANALYSIS

Name	p-value range	# Molecules
**Liver Hyperplasia/Hyperproliferation**	4.09E-01 - 8.58E-10	161
**Liver Proliferation**	1.60E-02 - 1.60E-02	2
**Liver Hemorrhaging**	4.25E-02 - 4.25E-02	2
**Liver Regeneration**	2.28E-01 - 7.92E-02	2
**Liver Fibrosis**	5.13E-01 - 1.13E-01	4

Nephrotoxicity

Name	p-value range	# Molecules
**Glomerular Injury**	3.15E-01 - 1.03E-02	4
**Kidney Failure**	5.10E-01 - 1.03E-02	4
**Renal Inflammation**	1.00E00 - 1.03E-02	3
**Renal Nephritis**	1.00E00 - 1.03E-02	3
**Renal Transformation**	1.03E-02 - 1.03E-02	1

Top Regulator Effect Networks

Top Networks

ID	Associated Network Functions	Score
**1**	Cancer, Organismal Injury and Abnormalities, Cell Morphology	62

(c) 2000-2018 QIAGEN. All rights reserved.

**2**	Cellular Assembly and Organization, Nervous System Development and Function, Cellular Movement	53
**3**	Cell Morphology, Cellular Assembly and Organization, Cellular Development	43
**4**	Connective Tissue Development and Function, Skeletal and Muscular System Development and Function, Tissue Development	38
**5**	Cellular Assembly and Organization, Cellular Function and Maintenance, Cellular Development	27

Top Tox Lists

Name	p-value	Overlap
**Renal Glomerulus Panel (Human)**	1.29E-02	11.8 % 2/17
**PPAR/RXR Activation**	4.56E-02	2.6 % 5/189
**Hormone Receptor Regulated Cholesterol Metabolism**	7.92E-02	12.5 % 1/8
**Vasopressin-induced Genes in Inner Medullary Renal Collecting Duct Cells (Rat)**	7.92E-02	12.5 % 1/8
**Renal Ischemic Resistance Panel (Rat)**	9.80E-02	10.0 % 1/10

(c) 2000-2018 QIAGEN. All rights reserved.

INGENUITY PATHWAY ANALYSIS

Top My Lists

Top My Pathways

Top Analysis-Ready Molecules

Phospho Fold Change

Molecules	Expr. Value	Chart
MAP1B*	↑ 1176.258	
KCNA2	↑ 625.375	
CAMK2G	↑ 246.111	
PDS5B	↑ 236.642	
SLC44A2	↑ 236.610	
MAP1A*	↑ 183.998	
NAV1	↑ 173.635	
TBR1*	↑ 164.941	
NEXN	↑ 145.514	
CAMK2B	↑ 130.937	

Phospho Fold Change

Molecules	Expr. Value	Chart
SFR1*	↓ -4141.815	

(c) 2000-2018 QIAGEN. All rights reserved.

**ARHGEF2**	↓ -1843.821	
**CDH6**	↓ -417.341	
**SPEG**	↓ -398.273	
**KNDC1**	↓ -393.838	
**HUWE1***	↓ -329.694	
**Ubap2l***	↓ -307.534	
**NSMF**	↓ -269.101	
**EEF1G**	↓ -238.029	
**BASP1***	↓ -169.079	

(c) 2000-2018 QIAGEN. All rights reserved.
