# Supplementary material for: Transcriptome-(phospho)proteome characterization of brain of a germline model of cytoplasmic-predominant Pten expression with autism-like phenotypes
Source: NPJ Genom Med. 2021 Jun 2;6:42. doi: 10.1038/s41525-021-00201-z (PMC8173008; doi:10.1038/s41525-021-00201-z)
Supplement: Supplementary file 4 — Reporting Summary [file 41525_2021_201_MOESM4_ESM.pdf]

nature research

Corresponding author(s): Charis Eng MD, PhD

Last updated by author(s): 03-01-2021

Reporting Summary

Nature Research wishes to improve the reproducibility of the work that we publish. This form provides structure for consistency and transparency in reporting. For further information on Nature Research policies, see our Editorial Policies and the Editorial Policy Checklist.

Statistics

For all statistical analyses, confirm that the following items are present in the figure legend, table legend, main text, or Methods section.

n/a	Confirmed	
☐	☒	The exact sample size (n) for each experimental group/condition, given as a discrete number and unit of measurement
☐	☒	A statement on whether measurements were taken from distinct samples or whether the same sample was measured repeatedly
☐	☒	The statistical test(s) used AND whether they are one- or two-sided<br>*Only common tests should be described solely by name; describe more complex techniques in the Methods section.*
☒	☐	A description of all covariates tested
☐	☒	A description of any assumptions or corrections, such as tests of normality and adjustment for multiple comparisons
☐	☒	A full description of the statistical parameters including central tendency (e.g. means) or other basic estimates (e.g. regression coefficient) AND variation (e.g. standard deviation) or associated estimates of uncertainty (e.g. confidence intervals)
☐	☒	For null hypothesis testing, the test statistic (e.g. F, t, r) with confidence intervals, effect sizes, degrees of freedom and P value noted<br>*Give P values as exact values whenever suitable.*
☒	☐	For Bayesian analysis, information on the choice of priors and Markov chain Monte Carlo settings
☒	☐	For hierarchical and complex designs, identification of the appropriate level for tests and full reporting of outcomes
☒	☐	Estimates of effect sizes (e.g. Cohen's d, Pearson's r), indicating how they were calculated

Our web collection on statistics for biologists contains articles on many of the points above.

Software and code

Policy information about availability of computer code

Data collection	No, code was used during the collection of data.
Data analysis	The majority of data analysis was carried out using open source software, which are outlined comprehensively in the methods sections, including String (string-db.org/), Reactome (reactome.org), and Cytoscape 3.7.0 (cytoscape.org). We also used the proprietary program from Qiagen called Ingenuity Pathway Analysis (qiagen.com/ingenuity).

For manuscripts utilizing custom algorithms or software that are central to the research but not yet described in published literature, software must be made available to editors and reviewers. We strongly encourage code deposition in a community repository (e.g. GitHub). See the Nature Research guidelines for submitting code & software for further information.

Data

Policy information about availability of data

All manuscripts must include a data availability statement. This statement should provide the following information, where applicable:

- Accession codes, unique identifiers, or web links for publicly available datasets
- A list of figures that have associated raw data
- A description of any restrictions on data availability

The complete raw data and analysis results are included in the manuscript or in the supplementary material. Figure 1 and Table 1 have associated raw data contained in Supplementary Data 1 file.

Field-specific reporting

Please select the one below that is the best fit for your research. If you are not sure, read the appropriate sections before making your selection.

☒ Life sciences ☐ Behavioural & social sciences ☐ Ecological, evolutionary & environmental sciences

For a reference copy of the document with all sections, see nature.com/documents/nr-reporting-summary-flat.pdf

Life sciences study design

All studies must disclose on these points even when the disclosure is negative.

Sample size	Every experiment in our study uses an N of 3 biological replicates.
Data exclusions	No data were excluded from our analysis.
Replication	Our data were implicitly replicated by our prior molecular and cellular phenotyping efforts, which are cited in the manuscript. Otherwise, no direct replication of this work is contained in our study.
Randomization	No randomization was utilized in this study. Our mutant animals were obtained on an as needed basis as it can be difficult to obtain enough of our mutant mice at the timepoints studied.
Blinding	No blinding was used in our study as it is not relevant to our experimental design or analysis.

Reporting for specific materials, systems and methods

We require information from authors about some types of materials, experimental systems and methods used in many studies. Here, indicate whether each material, system or method listed is relevant to your study. If you are not sure if a list item applies to your research, read the appropriate section before selecting a response.

Materials & experimental systems

n/a	Involved in the study	
☒	☐	Antibodies
☒	☐	Eukaryotic cell lines
☒	☐	Palaeontology and archaeology
☐	☒	Animals and other organisms
☒	☐	Human research participants
☒	☐	Clinical data
☒	☐	Dual use research of concern

Methods

n/a	Involved in the study	
☒	☐	ChIP-seq
☒	☐	Flow cytometry
☒	☐	MRI-based neuroimaging

Animals and other organisms

Policy information about studies involving animals; ARRIVE guidelines recommended for reporting animal research

Laboratory animals	We used mice from an outbred CD1 background. All mice were male. We used two timepoints, P14 and P40. This study did not involved experimentation on any live animals.
Wild animals	Not applicable
Field-collected samples	not applicable
Ethics oversight	Our use of animals was approved by our IACUC under protocol numbers 2018-1952 and 2017-1879. Our research was guided by the principles of laboratory animal care formulated by the national society for medical research.

Note that full information on the approval of the study protocol must also be provided in the manuscript.
